# Genome-wide exonic small interference RNA-mediated gene silencing regulates sexual reproduction in the homothallic fungus *Fusarium graminearum*

**DOI:** 10.1371/journal.pgen.1006595

**Published:** 2017-02-01

**Authors:** Hokyoung Son, Ae Ran Park, Jae Yun Lim, Chanseok Shin, Yin-Won Lee

**Affiliations:** 1 Center for Food and Bioconvergence, Seoul National University, Seoul, Republic of Korea; 2 Department of Agricultural Biotechnology, Seoul National University, Seoul, Republic of Korea; Oregon State University, UNITED STATES

## Abstract

Various ascomycete fungi possess sex-specific molecular mechanisms, such as repeat-induced point mutations, meiotic silencing by unpaired DNA, and unusual adenosine-to-inosine RNA editing, for genome defense or gene regulation. Using a combined analysis of functional genetics and deep sequencing of small noncoding RNA (sRNA), mRNA, and the degradome, we found that the sex-specifically induced exonic small interference RNA (ex-siRNA)-mediated RNA interference (RNAi) mechanism has an important role in fine-tuning the transcriptome during ascospore formation in the head blight fungus *Fusarium graminearum*. Approximately one-third of the total sRNAs were produced from the gene region, and sRNAs with an antisense direction or 5′-U were involved in post-transcriptional gene regulation by reducing the stability of the corresponding gene transcripts. Although both Dicers and Argonautes partially share their functions, the sex-specific RNAi pathway is primarily mediated by *Fg*Dicer1 and *Fg*Ago2, while the constitutively expressed RNAi components *Fg*Dicer2 and *Fg*Ago1 are responsible for hairpin-induced RNAi. Based on our results, we concluded that *F*. *graminearum* primarily utilizes ex-siRNA-mediated RNAi for ascosporogenesis but not for genome defenses and other developmental stages. Each fungal species appears to have evolved RNAi-based gene regulation for specific developmental stages or stress responses. This study provides new insights into the regulatory role of sRNAs in fungi and other lower eukaryotes.

## Author summary

Control of gene expression by small noncoding RNA (sRNA) has recently been highlighted as a significant post-transcriptional regulatory mechanism. To date, researchers have predominantly focused on the identification of microRNA-like RNAs (milRNAs) in fungi because microRNAs (miRNAs) are key regulators in animals and plants. In this study, we discovered that the sex-induced RNA interference (RNAi) mechanism had important roles in sexual reproduction in the head blight fungus *Fusarium graminearum*. In the late stages of sexual reproduction, small interference RNAs that were produced from gene regions (ex-siRNAs) were involved in post-transcriptional gene regulation at a genome-wide level. Based on our results, we concluded that *F*. *graminearum* specifically utilizes ex-siRNA-mediated RNAi for sexual development but not for other biological processes. This is the first genome-wide characterization of the sRNAs involved in fungal development.

## Introduction

The ascomycete fungus *Fusarium graminearum* is a major causative agent of Fusarium head blight (FHB) in small-grain cereals worldwide [1]. The fungus reproduces using sexual spores (ascospores) and asexual spores (conidia). Although both spore types contribute to disease initiation and propagation, ascospores serve as the primary inocula for FHB outbreaks because these spores are forcibly discharged into the air and can move long distances [2]. Additionally, the sexual development process ensures the production of survival structures required for overwintering [3] and the genetic diversity of the population [4]; therefore, understanding the molecular mechanisms underlying *F*. *graminearum* sexual reproduction is important for developing novel FHB disease-control strategies.

Perithecia (fruiting bodies) produced via sexual reproduction have complex multicellular structures with three distinguishable layers [5]. The perithecial outer layer composed of black and hard tissues/cells protects the inner components of the perithecia. Asci are biseriately arranged within perithecia and generate turgor pressure by accumulating osmolytes for forcible ascospore discharge [6]. Inside an ascus, a diploid zygote nucleus undergoes normal meiosis and mitosis to produce eight haploid nuclei, which are then sequestered into eight ascospores [7]. Various genetic and metabolic processes are intricately involved in sexual reproduction; these processes are likely fine-tuned temporally and spatially depending on each step of sexual development [5,8]. Many upstream signal transduction pathways and hundreds of signaling mediators, such as transcription factors and kinases, orchestrate sexual reproduction in *F*. *graminearum* [9–11].

RNA interference (RNAi) is a conserved gene-silencing mechanism that occurs at the post-transcriptional or post-translational level in eukaryotes, including fungi [12–14]. RNAi is triggered by small noncoding RNAs (sRNAs) of approximately 20–30 nucleotides, which are generally categorized as short interfering RNAs (siRNAs), microRNAs (miRNAs), and piwi-interacting RNAs (piRNAs); siRNAs and miRNAs have been extensively studied in fungi, although some piRNA-like sRNAs have been identified in *Neurospora crassa* [13,15]. The siRNA and miRNA duplexes are produced from double-stranded RNA (dsRNA) precursors that are cleaved by RNase III-family nuclease Dicers. The resulting siRNA or miRNA duplexes are loaded into an Argonaute protein of the RNA-induced silencing complex (RISC). Subsequent removal of the passenger strands of sRNA duplexes leads to the activation of the RISC, and this RISC-incorporated guide-strand sRNA is then used to identify complementary mRNA for silencing by either mRNA degradation or translational repression.

Elucidating the involvement of RNAi in quelling [16] and meiotic silencing by unpaired DNA (MSUD) [17,18] in *N*. *crassa* has been a major recent breakthrough in the genetics of filamentous fungi. Most fungal species possess functional RNAi components except for some single-cell yeasts [19]. Moreover, dsRNA-mediated gene silencing has been widely reported in fungi [13,20], which is promising for the development of an efficient gene-silencing method or the utilization of host-induced gene silencing for disease control [21,22]. Advanced next-generation sequencing technologies and bioinformatics tools have enabled researchers of non-model filamentous fungi to identify sRNAs. However, the biological roles of sRNAs in fungal development have rarely been identified, while RNAi pathways have crucial regulatory functions in cellular processes such as development, host defenses, mRNA processing, transcription, and translation in animals and plants [23–25]. Few sRNAs with important biological roles have been characterized in fungi, and sRNAs have been hypothesized to function under specific developmental or environmental conditions [26].

RNAi generally functions in genome defense from viruses and transposable elements in eukaryotes, including fungi [14,22]. The possible involvement of sRNAs in fungal development or responses to environmental stimuli has been proposed in *Aspergillus flavus* (responses to water activity and temperature) [27], *Magnaporthe oryzae* (fungal development) [28], and *Penicillium marneffei* (transition between mycelia and yeast phases) [29]. Previous studies have proposed biological roles for RNAi based on phenotypic defects of RNAi-deficient mutants. In the ascomycete fungus *Trichoderma atroviride*, different components of the RNAi machinery are involved in light-dependent asexual reproduction and light-independent hyphal growth [30]. Sexually induced RNAi machinery is required for sexual reproduction but not for virulence in the basidiomycete human pathogen *Cryptococcus neoformans* [31]. Components of the RNAi machinery involved in MSUD are also important for ascospore formation in *N*. *crassa* [18,32,33]. Null mutants of RNAi components showed various developmental defects, including dysfunction during sexual and asexual reproduction, in *Mucor circinelloides* [34]. In plant pathogenic fungi, important roles of pathogen-derived sRNAs in host-microbe interactions have been recently reported [35]. *Botrytis cinerea* microRNA-like RNAs (milRNAs) silence genes involved in host defense by inhibiting the host RNAi pathway [36]. However, there is little evidence from genome-wide approaches to characterize transcriptional reprograming by sRNAs during fungal development.

Our previous study revealed that the homothallic fungus *F*. *graminearum* possesses a functional MSUD mechanism, as is the case with the heterothallic fungus *N*. *crassa* [7]. The presence of MSUD led us to investigate the roles of sRNA-mediated RNAi in *F*. *graminearum* sexual development. A recent study revealed that hairpin RNA silenced target genes, and specific RNAi components were involved in this silencing process in *F*. *graminearum* [37]. Moreover, one of the Argonaute genes (*FgSMS2*/*FgAGO2*) was shown to be under the control of a mating-type gene and was required for sexual development in this fungus [9]. Thus, the objectives of the present study were to provide an in-depth characterization of the sexual phenotypes of RNAi component mutants and to determine the genome-wide correlation of sRNAs with the transcriptome and degradome during the sexual development of *F*. *graminearum*. This is the first genome-wide characterization of sRNAs involved in balancing transcript levels during fungal sexual development, and it provides insights into the role of sRNAs in fungi.

## Results

### Characterization of RNAi components

**Fig 1:**
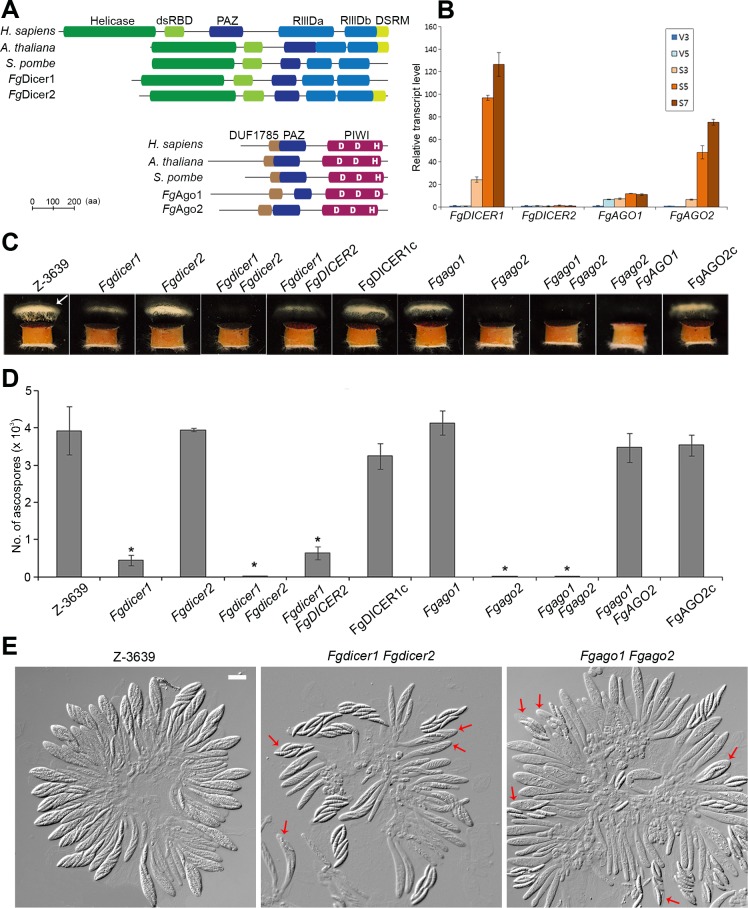
Characterization of Dicers and Argonautes in *F*. *graminearum*. (A) Domain architectures of *F*. *graminearum* Dicer and Argonaute proteins along with those of representative orthologs. Domains were predicted using SMART [38]. Conservation analysis of the Asp-Asp-His (DDH) motif that comprises the active sites of Argonautes. The DDH motif is conserved in *Fg*Ago2, but the third histidine residue is replaced by an aspartate in *Fg*Ago1. The amino acid sequences of orthologs: *Homo sapiens* Dicer1 (NP_803187), *Arabidopsis thaliana* Dcl2 (NP_566199), *Schizosaccharomyces pombe* Dcl1 (Q09884), *F*. *graminearum Fg*Dicer1 (Gene ID: FGSG_09025), *Fg*Dicer2 (Gene ID: FGSG_04408), *H*. *sapiens* Ago2 (NP_036286), *A*. *thaliana* Ago1 (NP_849784), *S*. *pombe* Ago1 (NP_587782), *F*. *graminearum Fg*Ago1 (Gene ID: FGSG_16976), and *F*. *graminearum Fg*Ago2 (Gene ID: FGSG_00348). (B) Expression profiles of *FgDICER1*, *FgDICER2*, *FgAGO1*, and *FgAGO2* in the *F*. *graminearum* wild-type strain during vegetative and sexual development. Transcript levels were analyzed via qRT-PCR during the vegetative (V3 and V5, 3 and 5 days after inoculation, respectively) and sexual stages (S3, S5 and S7, 3, 5, and 7 days after sexual induction, respectively) on carrot agar. The transcript level of the gene at the 3-day vegetative stage (V3) was arbitrarily set to 1, and this value was used for comparison to other periods. (C) Forcible ascospore discharge. A semi-circular agar block covered with perithecia was placed on a coverslip. Images were collected 48 h after the assay was initiated. White cloudy material (indicated with an arrow) represents discharged ascospores. (D) Number of discharged ascospores. Discharged ascospores were corrected for one week from the 7-day-old sexually induced cultures. (E) Asci rosettes. Imaging was performed 8 days after sexual induction. Red arrows indicate asci with defective ascospore delimitation. Scale bar = 20 μm.

*F*. *graminearum* possesses two Dicers and two Argonautes, as previously reported [37]. Both Dicers (*Fg*Dicer1 and *Fg*Dicer2) contain domain architectures similar to those of other representative orthologs; however, *Fg*Dicer1 and Dcl1 of *Schizosaccharomyces pombe* do not carry the double-stranded RNA-binding (DSRM) domain (Fig 1A). Each Argonaute has a conserved catalytic triad (Asp-Asp-His/Asp, DDH/D); *Fg*Ago2 contains the third histidine residue but is replaced by an aspartate in *Fg*Ago1. *FgAGO2* was also annotated as *FgSMS2* in a previous study [9].

We measured the transcript levels of these genes during vegetative growth and sexual development (Fig 1B). The expression levels of *FgDICER1* and *FgAGO2* were significantly increased after sexual induction, suggesting that they have active roles during perithecia development in *F*. *graminearum*. The transcript levels of *FgDICER2* were similarly maintained throughout the developmental stages, while *FgAGO1* transcripts accumulated to high levels as mycelia aged (V5, 5 days after inoculation) and as perithecia matured (S3–7, 3–7 days after sexual induction).

To characterize the roles of these genes in fungal development, we generated each gene deletion mutant using the homologous recombination method (S1 Fig and S1 Table). Although all of the tested *F*. *graminearum* strains produced mature perithecia with similar numbers, the *Fgdicer1* and *Fgago2* mutants were severely defective in forcible ascospore discharge, while the *Fgdicer2* and *Fgago1* mutants showed indistinguishable phenotypes compared to those of the wild type (Fig 1C and 1D). Except for defective sexual development, we could not find any defect in vegetative growth, virulence, or stress responses in the mutants, in accordance with a previous report [37].

Double-deletion mutants were generated to verify the redundant roles of Dicers or Argonautes. *Fgdicer2* and *Fgago1* mutants were first outcrossed with the Δ*mat2* strain to produce the HK338 (Δ*mat2* Δ*Fgdicer2*) and HK339 (Δ*mat2* Δ*Fgago1*) strains, respectively (S1 Table). Then, the double-deletion mutants HK340 (Δ*Fgdicer1* Δ*Fgdicer2*) and HK341 (Δ*Fgago1* Δ*Fgago2*) were generated from the outcrosses HK338 × *Fgdicer1* and HK339 × *Fgago2*, respectively. Deletion of both *FgDICER1* and *FgDICER2* almost completely abolished ascospore discharge, whereas *Fgdicer1* mutants discharged reduced amounts of ascospores compared to those of the wild type, suggesting a synergistic effect of mutant phenotypes between single- and double-deletion mutants (Fig 1C and 1D). Double-deletion *Fgago1 Fgago2* mutants showed similar phenotypes to those of *Fgago2* mutants with respect to forcible ascospore discharge.

Mutant phenotypes and synergisms were confirmed using several complementation assays (S2 Fig). First, reintroduction of the intact *FgDICER1* and *FgAGO2* genes into each corresponding single deletion mutant restored defective sexual development (Fig 1C and 1D). Next, the roles of *FgDICER2* and *FgAGO2* were further verified by introducing each gene into the double-deletion mutants using the outcrosses Δ*mat2* × HK340 (Δ*Fgdicer1* Δ*Fgdicer2*) and Δ*mat2* × HK341 (Δ*Fgago1* Δ*Fgago2*). The resulting strains (Δ*Fgdicer1 FgDICER2* and Δ*Fgago1 FgAGO2* in Fig 1C and 1D) showed similar phenotypes to those of the *Fgdicer1* and *Fgago1* mutants.

To determine the mechanism underlying defective ascospore discharge, we dissected mature perithecia to observe rosette asci (Fig 1E). The *F*. *graminearum* wild-type strain produced eight normal spindle-shaped ascospores per ascus. However, some asci of the mutants that were defective in ascospore discharge (Δ*Fgdicer1*, Δ*Fgago2*, Δ*Fgdicer1* Δ*Fgdicer2*, and Δ*Fgago1* Δ*Fgago2*) contained abnormally shaped ascospores and fewer than eight per ascus. The morphologies of abnormal-shaped ascospores vary, including larger ones, smaller ones, and broken ones. Moreover, small, ghost-like ascospores with incomplete spore delimitation were observed in these mutants (Fig 1E). Thus, we concluded that defective forcible ascospore discharge of Dicer or Argonaute mutants was due to abnormal ascospore production.

### Transcriptome analysis of RNAi component mutants

To investigate the molecular mechanisms underlying defective ascosporogenesis of the RNAi component mutants, we analyzed the transcriptomes of the *F*. *graminearum* strains using RNA-seq. Based on a threshold of reads per kilobase of exon per hundred million mapped reads (RPKHM) values (≥ 10) under all tested conditions, 11,908 of 13,820 genes were selected for further analyses (S2 Table). Differentially expressed genes (DEGs) were identified as genes displaying a greater than 3-fold change in transcript levels compared to those of the wild type.

**Fig 2:**
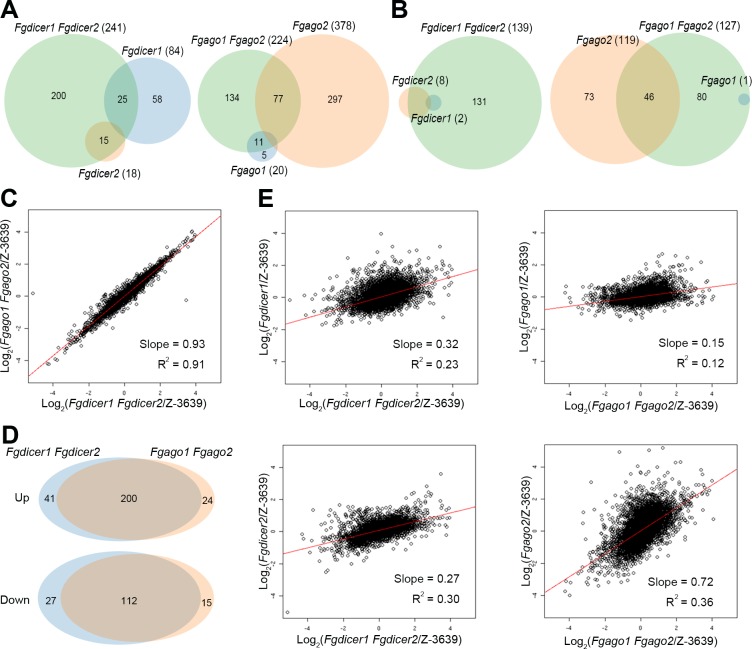
Transcriptome analysis of RNAi component mutants. Venn diagrams illustrating the overlap between upregulated (A) and downregulated (B) genes in the RNAi component mutants compared to those of the wild type. DEGs were identified as genes showing a greater than 3-fold change in transcript levels compared to those of the wild type. (C) Correlation analyses of transcriptomes of *Fgdicer1 Fgdicer2* and *Fgago1 Fgago2*. (D) Venn diagrams illustrating the overlap between DEGs of the *Fgdicer1 Fgdicer2* and *Fgago1 Fgago2* mutants. (E) Correlation analyses of transcriptomes of *Fgdicer1 Fgdicer2* and *Fgdicer1*, *Fgdicer1 Fgdicer2* and *Fgdicer2*, *Fgago1 Fgago2* and *Fgago1*, and *Fgago1 Fgago2* and *Fgago2*.

Deletion of both *FgDICER1* and *FgDICER2* induced the differential expression of 380 genes; 241 genes were upregulated, and 139 genes were downregulated (Fig 2A and 2B). While most of the upregulated genes in the *Fgdicer2* mutant overlapped with those of the *Fgdicer1 Fgdicer2* mutant (15/18), 70% of the genes (58/84) were specifically upregulated in the *Fgdicer1* mutant (Fig 2A). Similarly, 80% (297/378) and 61% of the genes (73/119) were increased or decreased, respectively, in their expression only in the *Fgago2* mutant but not in the *Fgago1 Fgago2* mutant (Fig 2A and 2B). In contrast, most of the downregulated genes in each Dicer single mutant were also reduced in transcript levels in the *Fgdicer1 Fgdicer2* mutant (Fig 2B).

Transcriptomes between the double-deletion mutants, *Fgdicer1 Fgdicer2* and *Fgago1 Fgago2*, were highly positively correlated (R^2^ = 0.91; Fig 2C), and most DEGs overlapped in these mutants (Fig 2D), suggesting that these two mutants were impaired in the same regulatory pathway. However, each transcriptome of *Fgdicer1* and *Fgdicer2* showed a mild positive correlation with that of *Fgdicer1 Fgdicer2*, supporting the functional synergism of *Fg*Dicer1- and *Fg*Dicer2-mediated transcriptional regulation during ascospore formation in *F*. *graminearum* (Fig 2E). While the transcriptome of the *Fgago1* mutant showed a relatively lower positive correlation with that of the *Fgago1 Fgago2* mutant, transcriptomes between the *Fgago2* and *Fgago1 Fgago2* mutants had a much higher correlation (slope = 0.72 and R^2^ = 0.36). Taken together, these results showed that the two Dicers and two Argonautes have redundant roles in the same biological pathway affecting ascospore formation in *F*. *graminearum*.

**Fig 3:**
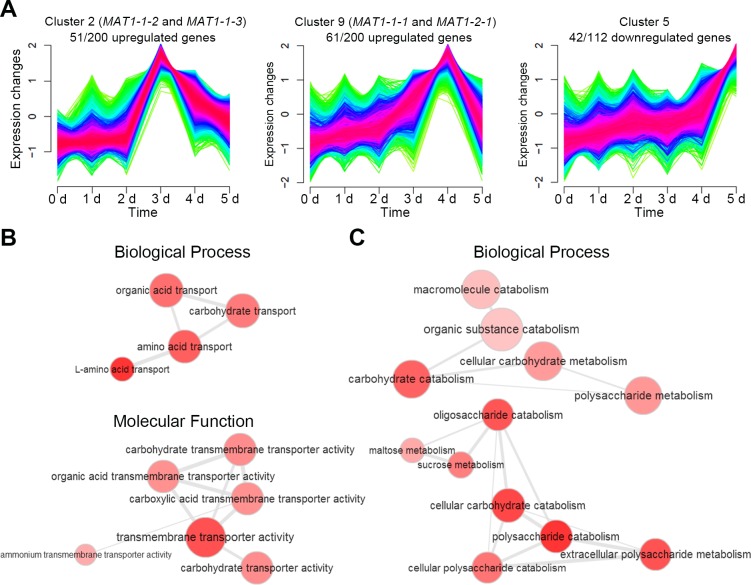
Characterization of DEGs in RNAi-deficient mutants. (A) Expression profiles of clustered groups including the mating-type genes. Fuzzy clustering categorized total genes into 10 groups depending on their expression profiles during sexual development (0–5 day after sexual induction). The genes included in groups 2 and 9 showed similar expression patterns as those of the mating-type genes (*MAT1-1-1*, *MAT1-1-2*, *MAT1-1-3*, and *MAT1-2-1*) during sexual reproduction. Transcriptome data during sexual development were obtained from a previous study [8] and re-analyzed for this study. (B) Gene Ontology (GO) enrichment network of the upregulated genes in the RNAi-deficient mutants. (C) GO enrichment network of the downregulated genes in the RNAi-deficient mutants. GO terms were statistically analyzed using GOstats [40] and visualized using REVIGO [41].

To dissect the characteristics of DEGs via disruption of the RNAi pathway, we examined the expression profiles of the upregulated (200) and downregulated (112) genes in both the *Fgdicer1 Fgdicer2* and *Fgago1 Fgago2* mutants compared to those of the wild type. Raw RNA-seq data from *F*. *graminearum* sexual developmental stages were obtained from a previous study [8], and the time-course expression patterns during sexual development were clustered into 10 groups using the R package *Mfuzz* [39] with the default setting, which performs fuzzy c-means clustering (S3 Fig and S3 Table). Mating-type genes (*MAT*s) are important sex-specific upstream regulators that orchestrate sexual reproduction processes in *F*. *graminearum*, and the RNAi pathway was proposed to be a downstream regulatory mechanism of *MAT*s [9]. *MAT1-1-2* and *MAT1-1-3* were included in group 2, and *MAT1-1-1* and *MAT1-2-1* were members of group 9 (Fig 3A and S3 Table). More than half of the upregulated genes were included in groups 2 or 9, in which the transcript levels were decreased at 4 days after sexual induction (Fig 3A and S4 Table). Group 5 possessed approximately 40% of the downregulated genes, and the transcript levels of group 5 genes were generally increased 4 days after sexual induction (Fig 3A). In short, the RNAi pathway affects the transcript abundance of genes closely related to the *MAT*-mediated regulatory mechanism during the late stages of sexual development in *F*. *graminearum*.

Our transcriptome analysis revealed that the RNAi pathway affects several important sexual reproduction-related molecular processes in *F*. *graminearum* (S2 Table). Pheromone precursor gene *PPG1* (Gene ID: FGSG_05061), two putative transcription factors (Gene ID: FGSG_01366 and FGSG_16753), and transaldolase (Gene ID: FGSG_13162), which are known to be important for sexual development, were significantly downregulated in RNAi-deficient mutants [9,10,42]. Moreover, three of these genes (FGSG_01366, FGSG_13162, and FGSG_16753) are under the control of *MAT* genes in *F*. *graminearum* [9]. In addition, 11 fungal-specific transcription factors with a Zn(II)_2_Cys_6_ fungal-type DNA-binding domain **(**FGSG_01760, FGSG_03892, FGSG_03912, FGSG_04782, FGSG_04786, FGSG_09111, FGSG_11186, FGSG_11358, FGSG_11672, FGSG_12597, and FGSG_16075) were downregulated in RNAi component mutants; null mutants of these factors did not show any mutant phenotype [10].

Gene Ontology (GO) enrichment analysis was additionally applied to classify the functions of the predicted DEGs, and the GO terms were statistically analyzed using GOstats [40] and subsequently visualized using REVIGO [41]. REVIGO provides a network structure of non-redundant GO terms (Fig 3B and 3C). Although we did not find any functionally characterized genes from the upregulated DEGs, they were assigned to 62 categories; 27 were included in biological processes, 26 in molecular functions, and 9 in cellular components. Accordingly, the most significant GO terms were related to the transport of organic compounds such as “amino acid transport” and “carbohydrate transport” of biological processes and "transmembrane transporter activity" of molecular functions (Fig 3B). Downregulated genes were assigned to 76 categories (36 in biological processes, 36 in molecular functions, and 4 in cellular components). Most GO terms corresponding to negatively regulated genes were involved in carbohydrate catabolism, such as “carbohydrate catabolism” and “polysaccharide catabolism” (Fig 3C).

Based on our GO enrichment analysis, we demonstrated that one of the important molecular processes regulated by the RNAi pathway is carbon metabolism. Production of triacylglycerol is important for perithecia development, and therefore, dynamic changes in carbon metabolism should occur during sexual reproduction processes in *F*. *graminearum* [43]. Accordingly, acetyl coenzyme A production and translocation between cellular organelles are closely involved in various steps of sexual development in *F*. *graminearum* [44–47]. In our transcriptome data, transaldolase (Gene ID: FGSG_13162) is involved in the pentose-phosphate pathway, and ascospore formation [9] was markedly downregulated in RNAi-deficient mutants (S2 Table).

### Characterization of sRNAs

To determine the relationship between sRNA production and transcriptome alteration, we isolated total low-molecular-weight RNAs from the wild-type and RNAi component mutant strains 5 days after sexual induction and used them for sRNA sequencing (S5 Table). After the adaptor sequences were trimmed, raw reads were normalized using DESeq to adjust for differences in library sizes [48]. Total reads with 18–32 nt perfect matches were used for alignment to the genome of *F*. *graminearum* [4,49].

**Fig 4:**
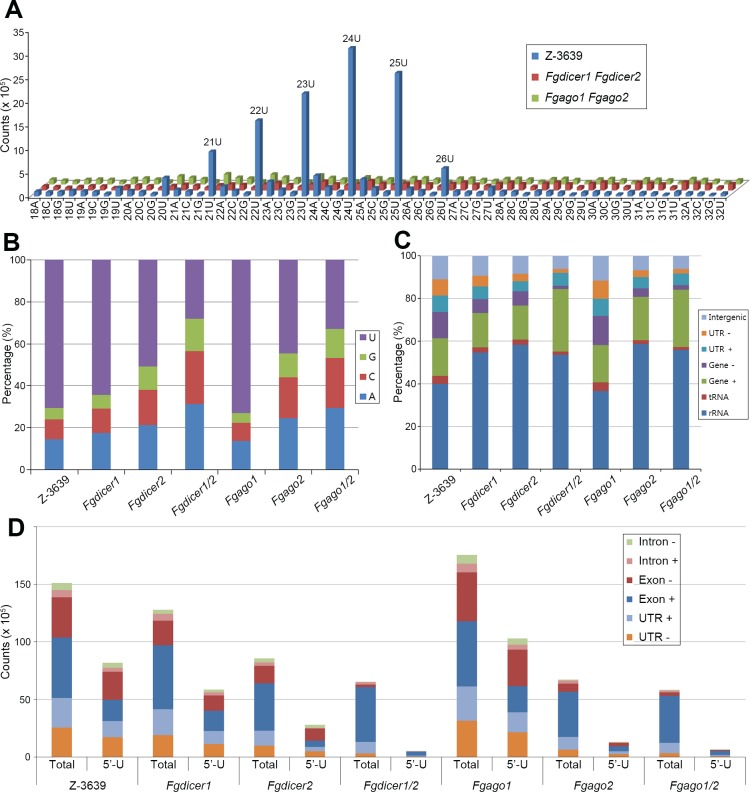
Characterization of sRNAs. (A) Nucleotide preference of 5′ end and size distribution of sRNAs produced by *F*. *graminearum* strains. (B) Analysis of 5′ end nucleotide preference of sRNAs produced by *F*. *graminearum* strains. (C) Relative abundance of sRNAs mapped to various genomic features produced by *F*. *graminearum* strains. (D) Absolute abundance of sRNAs mapped to various genomic features produced by *F*. *graminearum* strains. -, sRNAs with antisense direction; +, sRNAs with sense direction; 5′-U, sRNAs with 5′ uracil (22–25 nt).

The size distributions and 5′ end frequencies of sRNAs were analyzed. Identified sRNAs were 18–32 nt long, with a clear peak at approximately 24 nt in the *F*. *graminearum* wild-type strain (Fig 4A). The sRNAs with a 5′ end with U (5′-U) accounted for 70% of the total sRNAs in the wild-type strain and were mostly 21–26 nt long (Fig 4A and 4B). Deletion of either *FgDICER1*, *FgDICER2*, or *FgAGO2* attenuated the biases of sRNAs toward specific sizes (21–26 nt) and 5′-U; these changes were milder in *Fgdicer1* than those in *Fgdicer2* and *Fgago2*. Deletion of *FgAGO1* did not result in distinct changes in sRNA characteristics, but results for the *Fgago2* mutant were similar to those of *Fgdicer2* (Fig 4B). *Fgdicer1 Fgdicer2* and *Fgago1 Fgago2* mutants typically abolished these biases. Taken together, these findings showed that *F*. *graminearum* has distinct *Fg*Dicer1-, *Fg*Dicer2-, or *Fg*Ago2-dependent sRNA production/accumulation mechanisms and that these sRNAs have strong tendencies toward being 24 nt long and 5′-U. Moreover, both members of Dicers and Argonautes have functional redundancies.

To investigate the origin of sRNAs, we mapped sRNA sequences to the *F*. *graminearum* genome [4] with a recent annotation [49] to identify their various genomic features, such as coding sequences of rRNA, tRNA, and predicted proteins as well as intergenic regions (S6 Table). In the wild-type strain, sRNAs were in the rRNA region composed of approximately 40% of the total sRNAs (Fig 4C). Approximately 30% and 27% of the sRNAs mapped to protein-coding genes and intergenic regions, respectively. sRNA-producing loci frequently covered both protein-coding sequences and adjacent regions. Because there is little information about the untranslated region (UTR) in a recent annotation of *F*. *graminearum* [49], 500 bp upstream and downstream regions were arbitrarily denoted as 5′-UTR and 3′-UTR, respectively. Thereafter, 27% of the intergenic sequences were again separated into 16% of the UTRs and 11% of the intergenic sequences; predicted sRNAs corresponding to putative transcript sequences (protein-coding regions and UTRs) accounted for 46% (Fig 4C). Henceforth, we will call these sRNAs exonic siRNAs (ex-siRNAs), as previously reported [50].

When Dicer or Argonaute genes were deleted, the ratios of most genomic features aligned with sRNAs, except that the rRNA, protein-coding gene (sense strand), and UTR (sense strand), were decreased with analogous tendencies shown for size distributions and 5′-U in the corresponding deletion mutants (Fig 4C). In particular, sRNAs mapped to antisense sequences of putative transcripts were markedly reduced in the *Fgdicer1 Fgdicer2* and *Fgago1 Fgago2* mutants.

Various genomic features of sRNA-producing sequences were further assessed in detail with total normalized reads. The sRNAs produced from protein-coding genes converged on exons rather than introns (Fig 4D). The total amounts of sRNAs were decreased in the *Fgdicer1*, *Fgdicer2*, *Fgdicer1 Fgdicer2*, *Fgago2*, and *Fgago1 Fgago2* mutants, principally because of the reduced number of introns, exons, and UTRs with an antisense direction. Dicer- and Argonaute-dependent sRNAs with 5′-U (22–25 nt) were particularly enriched in antisense sequences of putative transcripts (Fig 4D and S6 Table). Moreover, the results confirmed that Dicers and Argonautes have redundant roles. Interestingly, total counts of sRNAs with antisense sequences of tRNA and rRNA were also markedly reduced in the *Fgdicer1 Fgdicer2* and *Fgago1 Fgago2* mutants, primarily because of a decreased number of sRNAs with 5′-U. The results suggest that Dicer- and Argonaute-dependent biogenesis/accumulation of sRNAs occurs at a genome-wide level.

### Characterization of sRNA-producing genes and regulatory functions of sRNAs

**Fig 5:**
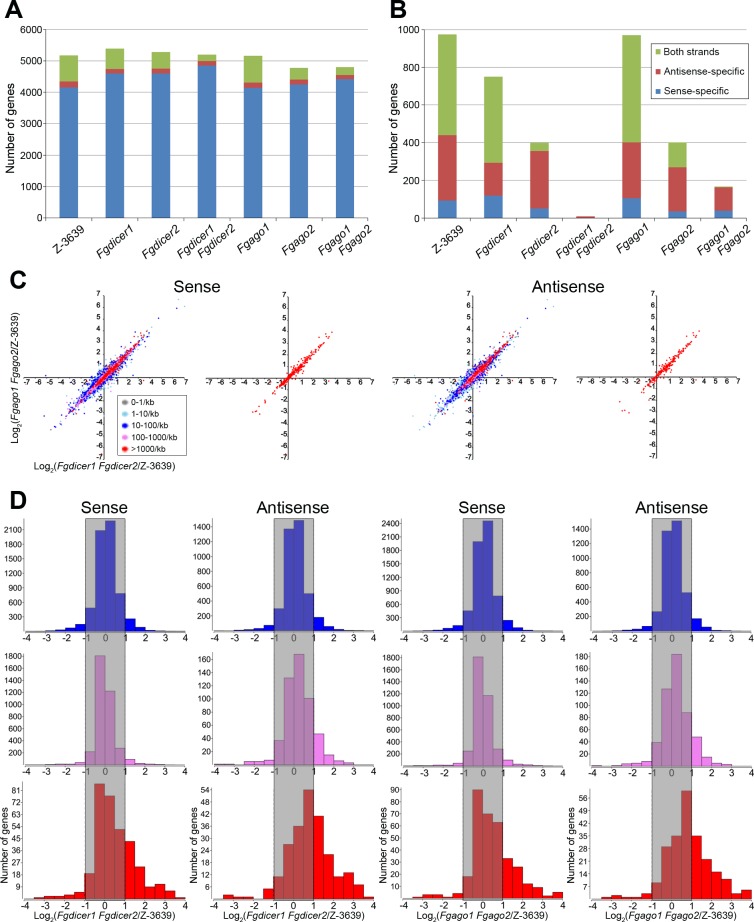
Characterization of sRNA-producing genes. (A) Number of genes that produce sRNAs depending on *F*. *graminearum* strains. (B) Number of genes that produce sRNAs with 5′-U depending on *F*. *graminearum* strains. (C) Correlation analyses of transcriptomes of *Fgdicer1 Fgdicer2* and *Fgago1 Fgago2* compared to that of the wild type depending on sRNA counts. The log_2_ ratio of transcript abundance in *Fgdicer1 Fgdicer2* versus wild-type (x axis) and *Fgago1 Fgago2* versus wild-type (y axis) is plotted. Colors indicate the sRNA density (reads per kilobase). (D) Correlation analyses of sRNA counts and transcript abundance. Gene numbers with corresponding log_2_ ratio of transcript abundance in *Fgdicer1 Fgdicer2* or *Fgago1 Fgago2* versus the wild-type strain Z-3639 were counted. Most genes producing antisense sRNAs more than 1000 counts per kilobase (red graphs) were positively regulated in *Fgdicer1 Fgdicer2* and *Fgago1 Fgago2* compared to those in the wild type. Colors indicate the sRNA density denoted in Fig 5C.

As most sRNAs were produced from gene regions, except for rRNA-mediated sRNAs, we focused on the characteristics of sRNA-producing genes (S7 Table). The *F*. *graminearum* wild-type strain produces sRNAs from 5180 genes, and 80% (4155/5180) of the genes produced only sense-specific sRNAs, suggesting that sense sRNAs were byproducts of mRNA degradation (Fig 5A and S8 Table). Accordingly, the number of genes producing only sense sRNAs was similar among the wild-type and RNAi component mutant strains.

We investigated 5′-U sRNA-producing genes from *F*. *graminearum* strains (Fig 5B and S8 Table). The genes were selected when the amount of 5′-U sRNA was more than 70% of the total sRNAs (S7 and S8 Tables). Approximately 1000 genes preferentially produced 5′-U sRNAs in the *F*. *graminearum* wild-type strain. Among them, more than half of the genes generated sRNAs with both orientations, and 10% of the genes produced only sense-specific sRNAs. A markedly reduced number of genes produced 5′-U sRNAs in *Fgdicer2* and *Fgago2* compared to those of the wild type, whereas the *Fgdicer1* mutant showed a slight reduction in the number of 5′-U sRNA-producing genes. Double deletion of *FgDICER1* and *FgDICER2* largely abolished 5′-U sRNA production. Genes producing antisense-specific or sense-specific sRNAs were counted in the *Fgago1 Fgago2* mutant. Taken together, these findings show that most sRNAs originating from the gene region with a 5′-U or antisense direction were distinctly produced in a Dicer-dependent manner.

We investigated whether sRNAs affected the transcript levels of corresponding genes in *F*. *graminearum*. First, all genes were categorized depending on the levels of sense or antisense sRNA counts, and they were applied in correlation analyses of transcript abundance between *Fgdicer1 Fgdicer2* and *Fgago1 Fgago2* versus the wild-type strain (Fig 5C). As the genes produced more sense or antisense sRNAs, a greater number of genes tended to accumulate more transcripts in *Fgdicer1 Fgdicer2* and *Fgago1 Fgago2* than those in the wild type. In particular, most genes producing either sense or antisense sRNAs at more than 1000 counts per kilobase (red dots in Fig 5C) were upregulated in the RNAi-deficient mutants. To analyze the negative correlation between sRNA production and transcript amounts of genes in detail, we assessed the gene numbers depending on transcript abundance in *Fgdicer1 Fgdicer2* or *Fgago1 Fgago2* versus the wild-type strain (Fig 5D). Whereas both sense and antisense sRNAs produced below 100 counts per kilobase did not show substantial changes in transcript levels of the corresponding genes, most genes producing antisense sRNAs at more than 1000 counts per kilobase (red graphs) were positively regulated in *Fgdicer1 Fgdicer2* and *Fgago1 Fgago2* compared to those in the wild type (Fig 5D). Both sense and antisense 5′-U sRNA (22–25 nt)-producing genes showed similar results to those obtained from antisense sRNAs (S4 Fig). Taken together, these findings show that a sRNA-mediated gene regulatory mechanism is not a critical factor that determines final transcript levels of most genes, but highly expressed sRNAs with antisense orientations participate in the negative transcriptional gene regulation of corresponding genes at a genome-wide level.

Although we found that *F*. *graminearum* possesses a global negative transcriptional regulatory mechanism involving antisense sRNAs during sexual development, only 57 genes produced antisense sRNAs among 200 upregulated DEGs (S2 and S7 Tables). Instead, a defective phenotype in ascospore production of RNAi-deficient mutants appears to be derived by the downregulation of sexual reproduction-related genes, such as *PPG1* (Gene ID: FGSG_05061), transcription factors (Gene ID: FGSG_01366 and FGSG_16753), and transaldolase (Gene ID: FGSG_13162), as noted above [9,10,42]. Thus, genes with substantial changes in transcript levels were not directly affected by RNAi-mediated gene regulation. Thirty-two transcription factor genes are possibly modulated by antisense sRNAs, and three of them (FGSG_01022, FGSG_09654, and *MAT1-2-1*) are closely related to perithecia development in *F*. *graminearum* [10]. In particular, RNAi participates in the regulation of one of the *MAT*s, *MAT1-2-1*, during sexual development. In the case of kinases, another important mediator for signal transduction pathways, four genes (FGSG_00469, FGSG_16383, FGSG_05418, and FGSG_16493) produced antisense sRNAs, and one of them (FGSG_05418) was shown to be important for ascospore formation in *F*. *graminearum* [11]. Although most of the mentioned genes involved in sexual development are known to be related to the perithecium development or the early stages of sexual development, our additional transcript analysis during sexual development demonstrated that they also have important functions during the late stages of sexual development (S5 Fig). In particular, most of tested genes were dynamically changed in expression approximately 5 days after sexual induction, and these patterns of several genes were altered in RNAi-deficient mutants compared to those of the wild type. Thus, the RNAi mechanism fine-tunes the transcriptome to modulate various molecular processes, including signal transduction networks, during ascospore production in *F*. *graminearum*.

### Validation of sRNA-seq and mRNA-seq

**Fig 6:**
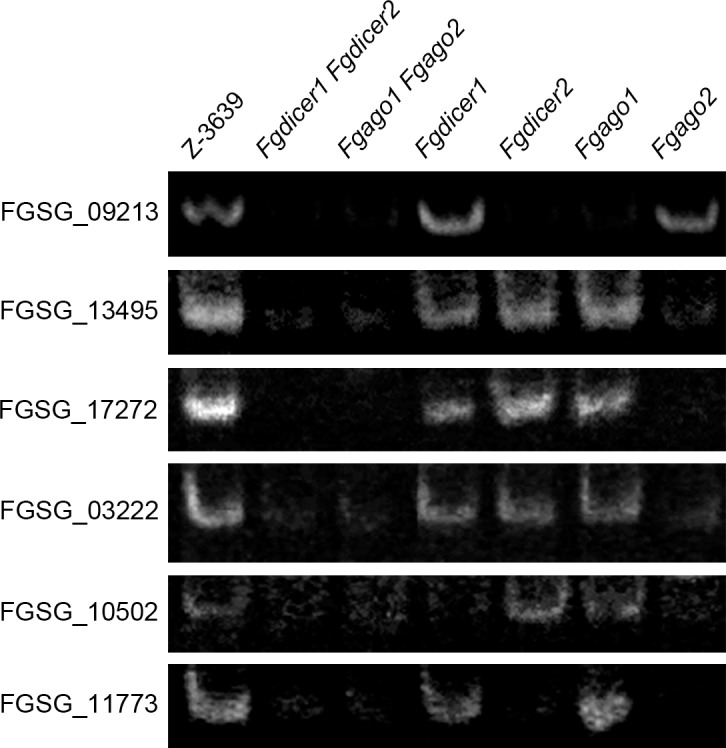
Detection of ex-siRNAs. Quantification of ex-siRNA candidates was performed using stem-loop RT-PCR assays. Each 100 ng of small RNA-enriched RNA samples was used for reverse transcription reactions, and the images were obtained from 25–30 cycles of PCR using 10% acrylamide gel.

**Fig 7:**
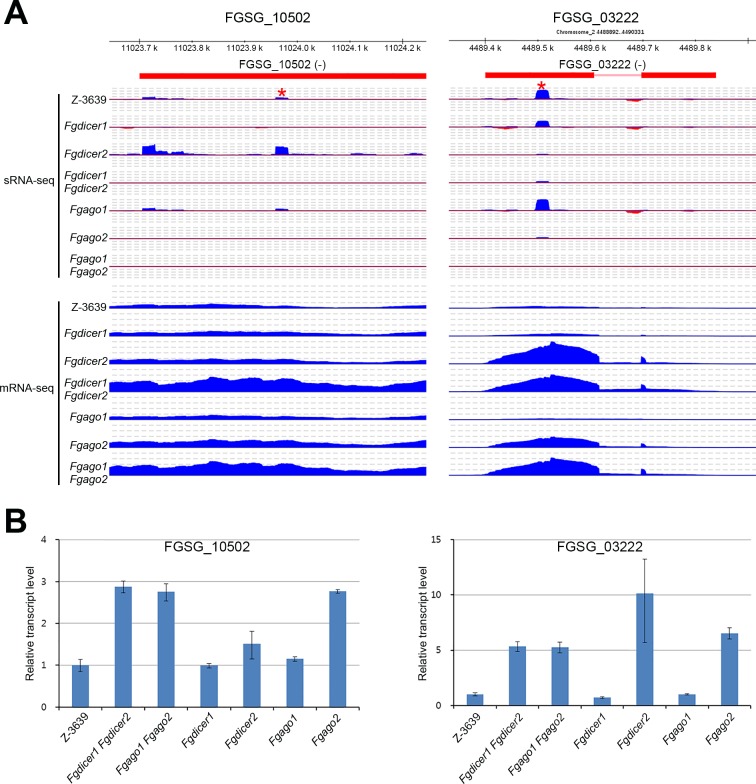
Representative IGV images of genes possibly regulated by ex-siRNA. (A) Aligned sRNA-seq and mRNA-seq results of *F*. *graminearum* strains were visualized using IGV. Red stars indicate selected ex-siRNA for RT-PCR. (B) Relative transcript abundances of genes in the *F*. *graminearum* strains during sexual development. Transcript levels were analyzed via qRT-PCR 5 days after sexual induction. The transcript level of the wild type was arbitrarily set to 1.

We visualized patterns of sequence alignments at the ex-siRNA-producing genes using Integrative Genomics Viewer (IGV) [51]. The IGV image of the gene (FGSG_11711) showed that ex-siRNAs are produced over the entire ranges of the gene (S6 Fig), demonstrating that total amount of siRNAs of the gene does not always reflect abundances of specific ex-siRNAs. Therefore, we identified ex-siRNAs with over 500 raw counts (S9 Table). Since repeated trials of the standard Northern blot analysis of six highly expressed ex-siRNAs (S7 Fig) were unsuccessful in detecting signals, we quantified the absolute amount of the ex-siRNA with the highest expression (siRNA of FGSG_09213) using stem-loop RT-PCR (S8 Fig). Whereas Northern blot analysis of our system could detect 1–10 fmoles synthetic siRNA of FGSG_09213, approximately 0.05 fmoles of siRNAs were detected in 100 ng of small RNA-enriched RNA samples from the *F*. *graminearum* wild type. Thereafter, we analyzed siRNAs using stem-loop RT-PCR (Fig 6). Abundances of siRNAs in the wild-type and the other mutant strains determined by RT-PCR were mostly consistent with our sRNA-seq results (S7 Fig and Fig 7A). We also verified that transcript levels of FGSG_10502 and FGSG_03222 were negatively correlated with corresponding ex-siRNA production (Fig 7B).

### Degradome analysis

**Fig 8:**
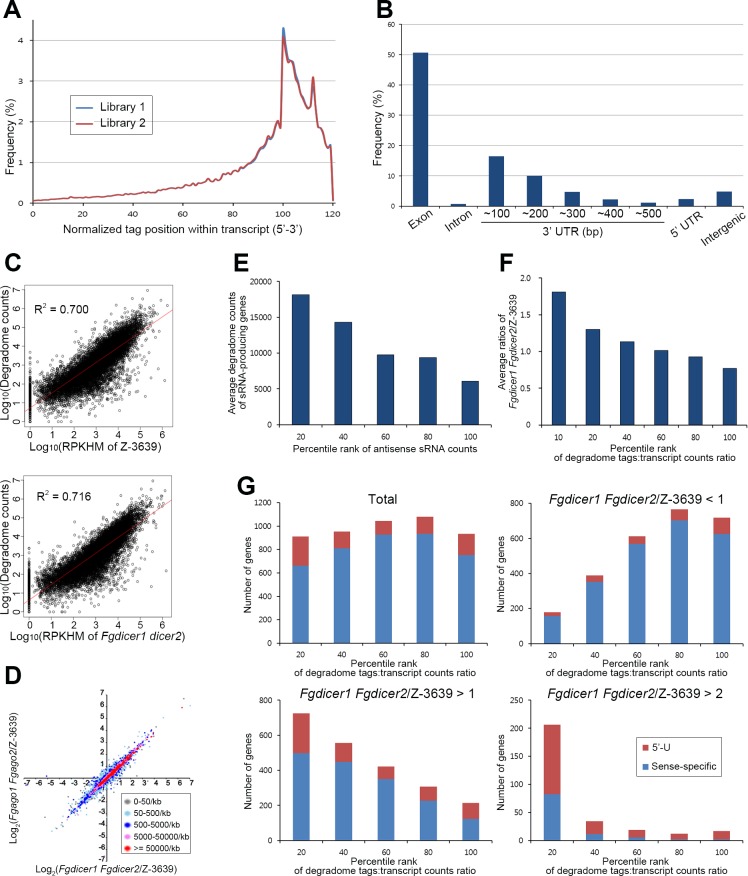
Degradome analyses. (A) Histogram displaying the 5′ positions of degradome tags from two wild-type libraries relative to normalized transcript positions. The protein-coding sequence of the transcript is exhibited from 0 to 100. (B) Frequency of degradome tags mapped to various genomic features produced by the wild-type strain. (C) Correlation analyses between wild-type degradome counts and transcript abundances of wild-type or *Fgdicer1 Fgdicer2* strains. (D) Correlation analyses of transcriptomes of *Fgdicer1 Fgdicer2* and *Fgago1 Fgago2* compared to that of the wild type. Colors indicate the degradome tag density (reads per kilobase). (E) Average degradome counts depending on percentile ranks of antisense sRNA counts. Percentile ranks of genes were assigned based on their antisense sRNA counts. (F) Average transcript abundance of *Fgdicer1 Fgdicer2* compared to the wild type depending on the percentile ranks of the degradome tags/transcript counts ratio. (G) Number of sRNA-producing genes corresponding to the percentile ranks of the degradome tags/transcript counts ratio. Numbers of sRNA-producing genes were divided into four groups depending on the transcript abundance of *Fgdicer1 Fgdicer2* compared to the wild type.

We analyzed the degradome of the *F*. *graminearum* wild-type strain to elucidate the mechanism for genome-wide sRNA-mediated gene silencing. Total RNAs were isolated from the wild-type strain 5 days after sexual induction, and RNA fragments with a 5′ monophosphate and polyA tail, which were cleaved products of transcripts, were subjected to the degradome library construction and deep sequencing. In our degradome sequencing method, we expected 16 nt reads after trimming the adapter sequences, and enriched reads of 16 nt and 17 nt were further used for alignment (S10 Table). Approximately 95% of the trimmed sequences from two independent degradome libraries perfectly matched one or more positions in the *F*. *graminearum* genome (S11 Table). To monitor transcript degradation, we used degradome tags with a sense direction for further analyses. The libraries of degradome tags were markedly biased toward the 3′ ends of protein coding sequences (CDS) and 3′-UTR regions (Fig 8A). Specifically, most degradome tags were enriched in the exon and 3′-UTR regions (Fig 8B and S11 Table).

The average of the total abundances of degradome tags for the CDS and 3′-UTR regions was calculated as reads per kilobase of sequence per two billion mapped sequence reads with a cutoff value of 10 (S12 Table). Comparison of the degradome abundance of the wild-type strain to the transcript values of the wild-type and RNAi component mutant strains similarly showed a distinct positive correlation (Fig 8C). Accordingly, genes with higher degradome counts tended to accumulate more transcripts in *Fgdicer1 Fgdicer2* and *Fgago1 Fgago2* than those in the wild type (Fig 8D). Taken together, these findings revealed that degradome tag abundance is primarily determined by the level of the corresponding gene transcript in *F*. *graminearum*.

The only known mechanism for siRNA-mediated gene silencing in filamentous fungi is siRNA-guided mRNA degradation [34]. As siRNA-guided endonucleolytic cleavage events should lead to the rapid decay of transcripts [52], we investigated the correlation between the abundance of degradome tags and antisense sRNA-mediated gene regulation. As genes produced more sRNAs with an antisense direction, the absolute number of degraded transcripts increased in the *F*. *graminearum* wild-type strain (Fig 8E). As the degradome generally reflects transcript levels, the abundances of degradome tags were normalized with transcript counts, and total genes were listed in the order of their given degradome tags:transcript counts ratios. When a higher portion of transcripts was degraded in the wild-type strain (higher percentile rank classes), corresponding genes tended to be upregulated in *Fgdicer1 Fgdicer2* mutants (Fig 8F), suggesting that Dicer-dependent gene regulation globally triggers transcript degradation in this fungus.

When sRNA-producing genes were evenly distributed among the percentile rank classes, markedly reduced and increased portions of genes produced sRNAs in the down- (*Fgdicer1 Fgdicer2*/Z-3639 < 1) and upregulated (*Fgdicer1 Fgdicer2*/Z-3639 > 1) gene groups, respectively (Fig 8G). More than 70% of the genes were concentrated in the top 20% of the percentile rank class in the genes that were upregulated more than 2-fold in *Fgdicer1 Fgdicer2* compared to that of the wild type (*Fgdicer1 Fgdicer2*/Z-3639 > 2; Fig 8G). Taken together, these results show that RNAi-mediated negative gene regulation occurs post-transcriptionally by degrading corresponding gene transcripts during sexual reproduction in *F*. *graminearum*.

## Discussion

Fungi have evolved several unique molecular mechanisms that are specifically activated during sexual reproduction processes [53]. Repeat-induced point mutation (RIP) and MSUD are well-known genome defense systems induced during meiotic cell division in some ascomycete fungi. In the heterothallic fungus *N*. *crassa*, RIP effectively detects and mutates repetitive transposable elements before meiotic prophase, resulting in the generation of nonfunctional transposons [54]. While RIP permanently mutates preexisting transposons, MSUD recognizes and suppresses the expression of repetitive sequences and therefore inhibits the mobilization of transposons during meiotic cell division [18]. Sex-induced silencing by RNAi was reported in the basidiomycete *C*. *neoformans*, which silences transgenes and transposons during sexual reproduction to protect the genome [31]. Recently, noncanonical adenosine-to-inosine RNA editing that enhances the diversity of gene products at the post-transcriptional level was shown to specifically occur during perithecium development in approximately half of the expressed genes in *F*. *graminearum* [55].

MSUD is also functional in *F*. *graminearum* despite its homothallic nature, but its activity was found to be much lower than that of *N*. *crassa* [7]. As MSUD is induced by RNAi-mediated gene silencing [26], *F*. *graminearum* was predicted to possess an effective RNAi, and a recent study reported that one of the Dicers (*Fg*Dicer2) and one of the Argonautes (*Fg*Ago1) were involved in hairpin-induced gene silencing during vegetative growth [37]. In the present study, we discovered that *F*. *graminearum* has a sexual specifically induced RNAi pathway that is important for ascospore formation. RNAi components, which are dispensable for hairpin-induced gene silencing during mycelial growth, *Fg*Dicer1 and *Fg*Ago2 primarily participate in the biogenesis of sRNAs with an antisense direction or 5′-U during sexual reproduction. Most sRNAs originated from transcript regions and globally affected expression of the corresponding genes at a post-transcriptional level by degrading corresponding transcripts.

Several types of fungal siRNAs have been characterized as functioning in genome defenses against transposable elements [56] or viruses [57] or for endogenous gene regulation to respond to heterochromatin formation [58] or DNA damage [59]. In particular, ex-siRNAs were proposed to regulate the target gene expression involved in fungal developmental processes in *M*. *circinelloides* and *T*. *atroviride* [30,50], while no correlation was found between ex-siRNAs and corresponding gene expression in *M*. *oryzae* [28]. In *F*. *graminearum*, RNAi components involved in ex-siRNA-mediated RNAi (*FgAGO2* and *FgDICER1*) were specifically functional during the late stages of sexual development. Similarly, large amounts of ex-siRNAs (approximately 30% of total sRNAs) were produced during sexual reproduction in *F*. *graminearum*, whereas only 2%, 4%, and 5% of sRNAs were produced from protein-coding regions in *T*. *atroviride* [30], *M*. *oryzae* [60], and *F*. *oxysporum* [61], respectively. RNAi-deficient mutants of *M*. *circinelloides* and *T*. *atroviride* were defective in asexual development, but those of *F*. *graminearum* and *M*. *oryzae* were not [37,60]. Instead, *F*. *graminearum* has evolved a sex-specifically induced ex-siRNA-mediated RNAi for genome-wide post-transcriptional gene regulation, which is important for ascosporogenesis.

More than half of the genes with decreasing or increasing tendencies in expression during ascospore formation in the *F*. *graminearum* wild-type strain were positively or negatively regulated in RNAi-deficient mutants, respectively (Fig 3A). In particular, 50% of the upregulated genes in the RNAi-deficient mutants showed similar expression patterns to those of *MAT* genes during perithecia development, demonstrating that RNAi is one of the key pathways under the control of *MAT* genes, as previously suggested [9]. However, ex-siRNA-mediated RNAi is not a critical process that determines absolute transcript amounts of most genes compared to other transcriptional or post-transcriptional regulatory mechanisms of this fungus. Not all ex-siRNA-producing genes were significantly downregulated in RNAi-deficient mutants, and sex-specific RNAi appears to be used for minute negative post-transcriptional gene regulation. Indeed, sex-specific RNAi orchestrates global gene regulation, which may alter signal transduction networks or carbon metabolism involved in sexual development in *F*. *graminearum* (Fig 3B).

In addition to a regulatory role for ascosporogenesis, sex-specific ex-siRNAs may not be closely involved in the genome defense mechanism. Few gene duplications and transposons have been found in the *F*. *graminearum* genome, although large amounts of siRNAs are produced during sexual reproduction [4]. In addition, ex-siRNAs of *F*. *graminearum* are different from sex-specific MSUD-associated siRNAs (masiRNAs). MSUD detects unpaired DNA regions during meiosis and leads to siRNA production for gene silencing in the heterothallic fungus *N*. *crassa* [26,62]. In a recent study, naturally unpaired regions between two mating partners were predicted to be major sources for ex-siRNAs in the heterothallic fungus *N*. *crassa* [63]. However, *F*. *graminearum* is homothallic, and therefore, there should be no unpaired DNA regions during meiotic cell division.

Two Dicers and two Argonautes have redundant and separate roles for RNAi in *F*. *graminearum*. Passenger strand degradation is generally required for RNAi function, and guide strands bound by Argonaute are protected by degradation [64,65]. Therefore, the lack of accumulation of sRNAs in *Fgago* mutants indicated that these sRNAs are functional for RNAi so that in its absence, the siRNAs would be rapidly degraded. Double-deletion mutants of both Dicers and Argonautes similarly abolished sRNA production and altered transcriptomes, suggesting that both of them have redundant functions (Figs 2 and 4). However, *Fg*Dicer2 has a more important role in global sRNA production than that of *Fg*Dicer1; *Fg*Dicer1 is mostly dispensable for hairpin-mediated sRNA production [37]. Intriguingly, *Fg*Ago2 has a major function in global sRNA production in *F*. *graminearum*, whereas *Fg*Ago1 is specifically involved in hairpin-induced sRNA production. Due to the redundant and separate roles of Argonautes, deletion of *FgAGO2* may lead to excessive binding of guide strand siRNA to FgAgo1, resulting in unexpected transcriptional regulation. For this reason, fewer genes were upregulated in the *Fgago1 Fgago2* double mutant than those in the *Fgago2* single mutant (Fig 2A). Similarly, Ago1 and Ago2 function in a partially redundant manner but generally have roles in miRNA function and siRNA-triggered RNAi, respectively, in *Drosophila melanogaster* [66].

The specificities of RNAi components may be derived by characteristics of a fungal RNAi system. Sad-1 (RdRp), Sms-3 (Dicer), Sms-2 (Argonaute), and other RNAi components form a silencing complex so that these RNAi components together govern a specific RNAi pathway involved in MSUD in *N*. *crassa* [67]. If this is the case in *F*. *graminearum*, at least two types of silencing complexes with different specificities may be involved in siRNA- or hairpin-mediated gene silencing; *Fg*Ago1 and *Fg*Dicer2 would be components of the same silencing complex for hairpin-mediated RNAi in *F*. *graminearum* [37]. The target specificities of Argonautes may also be a reason for the distinct functions of two Argonautes. In *A. thaliana*, *At*Ago2 and *At*Ago4 preferentially bind to sRNAs with a 5′ terminal adenosine, while *At*Ago1 predominantly recruits miRNAs with 5′-U among ten Ago proteins [68].

In the present study, using combined analysis of functional genetics and deep sequencing of sRNAs and the transcriptome and degradome, we demonstrated that the sex-specifically induced ex-siRNA-mediated RNAi mechanism fine-tunes the transcriptome for ascospore formation in *F*. *graminearum*. Ex-siRNA functions are important for various developmental stages and stress responses in the basal fungus *M*. *circinelloides*, but *F*. *graminearum* has evolved to utilize ex-siRNAs for a specific developmental stage. Therefore, ex-siRNA-mediated RNAi might be involved in various fungal developmental stages and stress responses depending on the fungal species and should be highlighted as an important post-transcriptional regulatory mechanism in fungi.

## Methods

### Fungal strains and media

The wild-type strain Z-3639 and transgenic strains derived from this strain were used in this study (S1 Table). Fungal strains were stored as conidia and mycelia in 30% glycerol solution at -80°C. All of the media used in this study were prepared as described in the *Fusarium* laboratory manual [1].

### Nucleic acid manipulation and quantitative real-time (qRT)-PCR

Genomic DNA was extracted from lyophilized mycelia according to the *Fusarium* laboratory manual [1]. Restriction endonuclease digestion, agarose gel electrophoresis, Southern and Northern blotting, and hybridization with ^32^P-labeled probes were performed following standard protocols [69]. PCR and qRT-PCR primers used in this study were synthesized by an oligonucleotide synthesis facility (Bionics, Seoul, Korea) (S13 Table). Stem-loop RT-PCR analyses of siRNAs were performed as previously described [70,71].

For detection of small RNAs, RNAs that are highly enriched for small RNA species (less than 200 nt) were isolated using a *mir*Vana^TM^ miRNA isolation kit (Invitrogen), separated in a 15% urea-polyacrylamide gel, and transferred to Immobilon-Ny + membranes (Millipore, Billerica, MA, USA). The membranes were probed with ^32^P-labeled oligonucleotides in PerfectHyb^TM^ Plus Hybridization Buffer (Sigma-Aldrich, St. Louis, MO, USA) at 37°C. Hybridization and washing steps were performed as previously described [72].

Total RNA was isolated from mycelia that were ground in liquid nitrogen using an Easy-Spin total RNA extraction kit (iNtRON Biotech, Seongnam, Korea), and each first-strand cDNA was synthesized using SuperScript III reverse transcriptase (Invitrogen, Carlsbad, CA, USA). qRT-PCR was performed with SYBR Green Supermix (Bio-Rad, Hercules, CA, USA) and a 7500 real-time PCR system (Applied Biosystems, Foster City, CA, USA) with corresponding primer pairs (S13 Table). The ubiquitin C-terminal hydrolase gene *UBH* (Gene ID: FGSG_01231) was used as a reference gene. We compared the cycle threshold (2^-ΔΔ*CT*^) to measure the transcript levels of target genes in different conditions. PCR was performed three times with three replicates per run.

### Targeted gene deletion and genetic complementation

The double-joint (DJ) PCR strategy was applied to construct fusion PCR products for targeted gene deletion and complementation [73]. For *FgDICER1* deletion, the 5′ and 3′ flanking regions of *FgDICER1* were amplified from the genomic DNA of the *F*. *graminearum* wild-type strain (S1 Fig). A geneticin resistance gene cassette (*GEN*) was amplified from pII99. Three amplicons (5′ flanking region, *GEN*, and 3′ flanking region) were mixed at a 1:3:1 molar ratio and fused by a second round of DJ PCR. Finally, the fusion constructs were amplified with the nested primers using the second-round PCR product as a template.

For complementation, the 5′ flanking region that included the *FgDICER1* open-reading frame with its own promoter and 3′ flanking region were amplified from genomic DNA of the wild-type strain. The *HYG* construct was amplified from pBCATPH. Three amplicons were then fused in a second round of DJ PCR. Finally, the fusion constructs for transformation were amplified with the nested primers using the second-round PCR product as a template. Fungal transformation was performed as previously described [44]. The same strategy was used for deletion and complementation of other RNAi component genes (S2 Fig).

### Sexual crosses

For self-crosses, mycelia were grown on carrot agar for 5 days and then removed with the back of a surgical blade (surgical blade #11; Feather Safety Razor, Osaka, Japan) while applying 2.5% sterilized Tween 60 solution [1]. For outcrosses, the heterothallic Δ*mat2* strain was fertilized with 1 ml of a conidial suspension (10^5^ conidia/ml) from fertilizing parents. All of the sexually induced cultures were incubated under near-UV light (wavelength: 365 nm; HKiv Import & Export Co., Ltd., Xiamen, China) at 25°C.

### Deep sequencing of transcriptome, sRNAs, and degradome

Transcriptome analysis in *F*. *graminearum* was performed as previously described [74]. Total RNA was isolated from each fungal culture at 5 days after sexual induction on carrot agar using an Easy-Spin total RNA extraction kit (iNtRON Biotech). More than five biological replicates of each strain were pooled for RNA-seq library construction. RNA-seq libraries were constructed using the Illumina TruSeq^TM^ RNA sample prep kit with no modifications to the standard low-throughput protocol. Samples were run on an Illumina HiSeq2000 instrument using the reagents provided in the Illumina TruSeq paired-end (PE) cluster kit V3-cBot-HS and the TruSeq SBS kit v3-HS (200 cycles). Similarly, a small RNA library was prepared using a TruSeq small RNA library prep kit following the manufacturer’s instruction. Then, each library was subjected to single-end 100 bp sequencing using a HiSeq2000 instrument.

Degradome-seq was performed as previously described with some modifications [75,76]. Poly (A)^+^ RNA was isolated using the NucleoTraP^®^ mRNA purification kits (Machery-Nagel, Düren, Germany) according to the manufacturer’s instructions. A 5′ RNA adapter (5′-GUUCAGAGUUCUACAGUCCGACGAUC-3′) was ligated to the cleavage products, which contain a 5′ monophosphate. The ligated products were reverse-transcribed into cDNA using an oligo (dT) primer (5′-CGAGCACAGAATTAATACGACTTTTTTTTTTTTTTTTTT-3′) by SuperScript III reverse transcriptase (Invitrogen) and amplified by PCR with a pair of cDNA primers (5´-GTTCAGAGTTCTACAGTCCGA-3′ and 5´-CGAGCACAGAATTAATACGACT-3′). The resulting product was digested with a MmeI (NEB, MA, USA) to obtain short fragments from the 5´ end of double-stranded cDNA. The digested products were ligated with an annealed duplex DNA adapter (top, 5´-p- TGGAATTCTCGGGTGCCAAGG- 3′ and bottom, 5´-CCTTGGCACCCGAGAATTCCANN-3′) using T4 DNA ligase (NEB). The ligated DNA products (~62 bp) were isolated using a 12% polyacrylamide gel (PAGE), and the purified products were amplified by PCR with a set of indexed TruSeq. The final PCR products were purified by running a 6% PAGE gel based on size (~128 bp). Most enzymes and primer sequences used in this study were obtained from a TruSeq small RNA library prep kit. Libraries were used for throughput sequencing on a HiSeq2000 platform.

Alignments were performed with BWA [77] using the *F*. *graminearum* genome [49], and the htseq-count script of the HTSeq package was used to compute the counts per gene [78]. Genome-wide transcript levels were quantified in reads per kilobase of exon per hundred million mapped sequence reads (RPKHM) [79]. Genes with maximum RPKHM values below 10 were deleted from the analysis. DEGs were identified based on fold-change values.

High-quality small RNA reads were obtained from raw reads by filtering out poor quality reads and removing adaptor sequences using the FASTX toolkit [80]. Adaptor-trimmed unique sequences were aligned to the *F*. *graminearum* genome using bowtie [49,81], and structural RNAs, such as tRNA, rRNA, snRNA, and snoRNA, were identified. The perfectly matched reads between 18–32 nt (for sRNAs) in length were selected.

### Data access

The data discussed in this publication have been deposited in NCBI's Gene Expression Omnibus [82] and are accessible through the accession numbers GSE87724 for mRNA-seq and GSE87835 for sRNA-seq. Raw data of degradome-seq have been deposited in NCBI's Sequence Read Archive (BioProject PRJNA348145).

## Supporting information

S1 Fig Targeted deletion of RNAi component genes. The deletion of *FgDICER1* (A), *FgDICER2* (B), *FgAGO1* (C), and *FgAGO2* (D) was achieved using homologous recombination. Deletion mutants were confirmed by Southern blot analysis. The sizes of the DNA standards (kb) used are indicated to the left of each blot. Southern blot: lane 1, *F*. *graminearum* wild-type strain Z-3639; lanes 2, 3, and 4, deletion mutants. *GEN*, genetic resistance gene cassette; *HYG*, hygromycin B resistance gene cassette; E, EcoRI; A, AvaI.
(TIF)

S2 Fig Complementation assays. Complementation experiments of *Fgdicer1* (A) and *Fgago2* (B) were performed, and the resulting strains were confirmed by Southern blot analysis. Lane 1, *F*. *graminearum* wild-type strain Z-3636; lane 2, a deletion mutant; lane 3, a complemented strain. The sizes of the DNA standards (kb) used are indicated to the left of each blot. E, EcoRI; P, C, ClaI.
(TIF)

S3 Fig Expression profiles of genes included in the clustered groups. Fuzzy clustering categorized total genes into 10 groups depending on their expression profiles during sexual development. RNA-seq results were obtained from a previous study and realigned for this analysis [8].
(TIF)

S4 Fig Characterization of sRNA-producing genes. (A) Correlation analyses of transcriptomes of *Fgdicer1 Fgdicer2* and *Fgago1 Fgago2* compared to the wild type depending on counts of 5′-U sRNAs (22–25 nt). The log_2_ ratio of transcript abundance in *Fgdicer1 Fgdicer2* versus wild-type (x axis) and *Fgago1 Fgago2* versus wild-type (y axis) is plotted. Colors indicate the sRNA density (reads per kilobase). (B) Correlation analyses between sRNA counts and transcript abundance. Gene numbers with corresponding log_2_ ratio of transcript abundance in *Fgdicer1 Fgdicer2* or *Fgago1 Fgago2* versus the wild-type strain Z-3639 were counted. Most genes producing antisense sRNAs at more than 1000 counts per kilobase (red graphs) were positively regulated in *Fgdicer1 Fgdicer2* and *Fgago1 Fgago2* compared to the wild type. Colors indicate the sRNA density.
(TIF)

S5 Fig Relative transcript accumulation of candidate genes responsible for sexual defects in *F*. *graminearum* strains. The transcript levels of each locus were analyzed by qRT-PCR in the wild-type, *Fgdicer1 Fgdicer2*, and *Fgago1 Fgago2* strains during the sexual stages (S1, S3, S5 and S7. 1, 3, 5, and 7 days after sexual induction, respectively) on carrot agar.
(TIF)

S6 Fig The IGV image of the FGSG_11711 gene. Aligned sRNA-seq results of *F*. *graminearum* strains were visualized using IGV.
(TIF)

S7 Fig The IGV image of the sRNA-producing genes. Aligned sRNA-seq results of *F*. *graminearum* strains were visualized using IGV.
(TIF)

S8 Fig The sensitivity of the Northern blot and stem-loop RT-PCR assay. (A) Gel blot analysis of siRNA of FGSG_09213 expression. Synthetic siRNA and 1 μg of the *F*. *graminearum* wild-type total RNA (WT) enriched in small RNA species were used to determine sensitivity of the Northern blot. (B) Standard curve of the siRNA. Stem-loop RT-PCR assay was used for the analysis. Red line indicates that 0.048 fmoles of siRNA were detected in 100 ng of the small RNA-enriched RNA samples of the *F*. *graminearum* wild-type strain.
(TIF)

S1 Table *F*. *graminearum* strains used in this study. (DOC)

S2 Table Total RNA-seq data of the wild-type and RNAi mutant strains. (XLS)

S3 Table Total RNA-seq data of the wild-type strain during sexual development. (XLS)

S4 Table Number of differentially expressed genes depending on cluster groups. (DOC)

S5 Table Statistical summary of sRNA sequencing raw data. (DOC)

S6 Table Statistical summary of aligned sRNA sequencing data. (DOC)

S7 Table sRNA-producing genes with sRNA counts. (XLS)

S8 Table Characterization of sRNA-producing genes. (DOC)

S9 Table Highly expressed ex-siRNA candidates. (XLSX)

S10 Table Size distribution of adaptor-trimmed degradome sequencing raw reads. (DOC)

S11 Table Statistical summary of degradome sequencing results and distribution of degradome tags mapped to the genome sequence of *F*. *graminearum*. (DOC)

S12 Table Degradome tags per gene region. (XLS)

S13 Table Primers used in this study. (DOC)
