# Supplementary figures and images for: Genome-wide exonic small interference RNA-mediated gene silencing regulates sexual reproduction in the homothallic fungus Fusarium graminearum
Source: PLoS Genet. 2017 Feb 1;13(2):e1006595. doi: 10.1371/journal.pgen.1006595 (PMC5310905; doi:10.1371/journal.pgen.1006595)

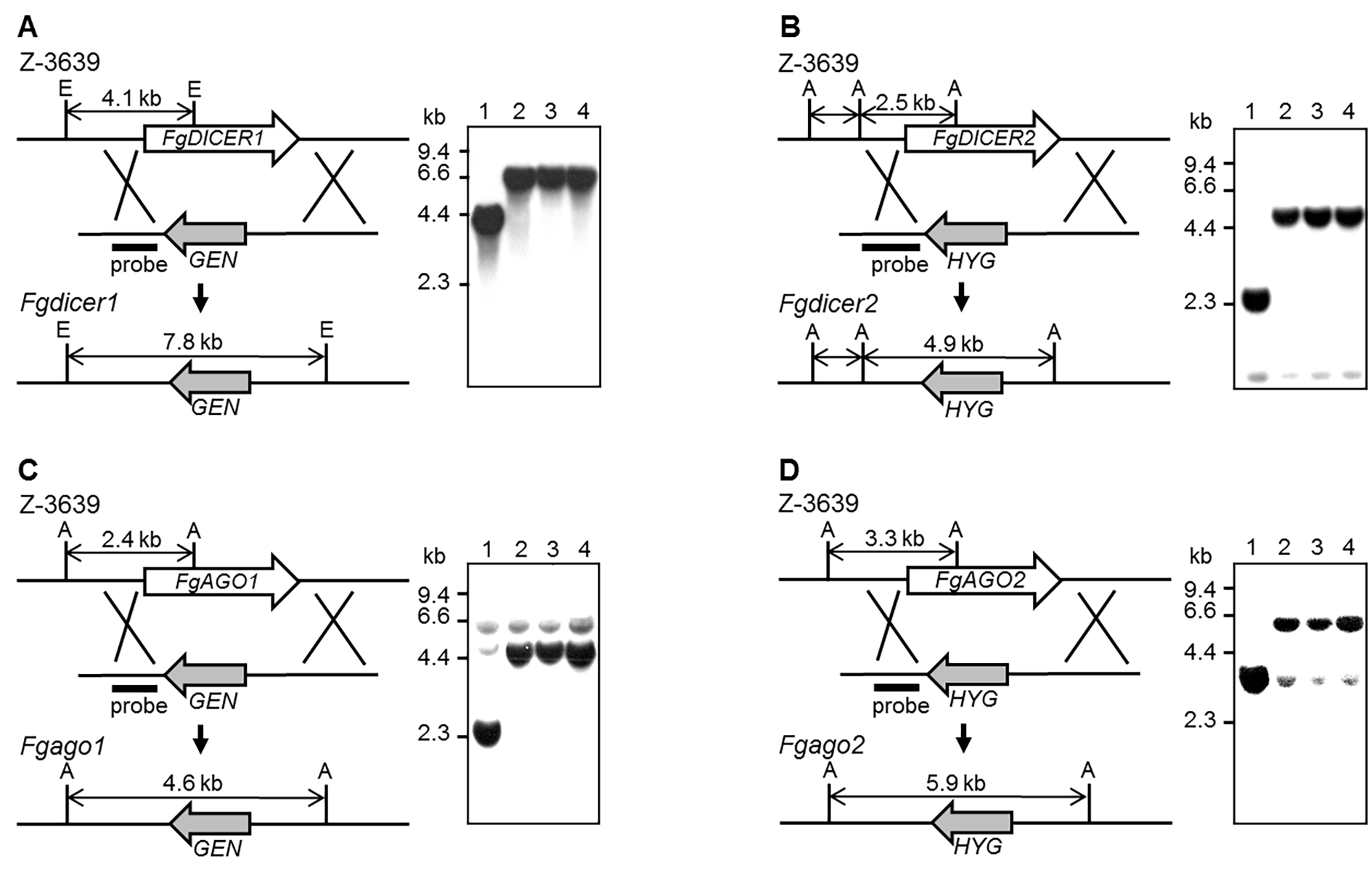

Supplement: S1 Fig — The deletion of FgDICER1 (A), FgDICER2 (B), FgAGO1 (C), and FgAGO2 (D) was achieved using homologous recombination. Deletion mutants were confirmed by Southern blot analysis. The sizes of the DNA standards (kb) used are indicated to the left of each blot. Southern blot: lane 1, F. graminearum wild-type strain Z-3639; lanes 2, 3, and 4, deletion mutants. GEN, genetic resistance gene cassette; HYG, hygromycin B resistance gene cassette; E, EcoRI; A, AvaI. (TIF) [file pgen.1006595.s001.tif]

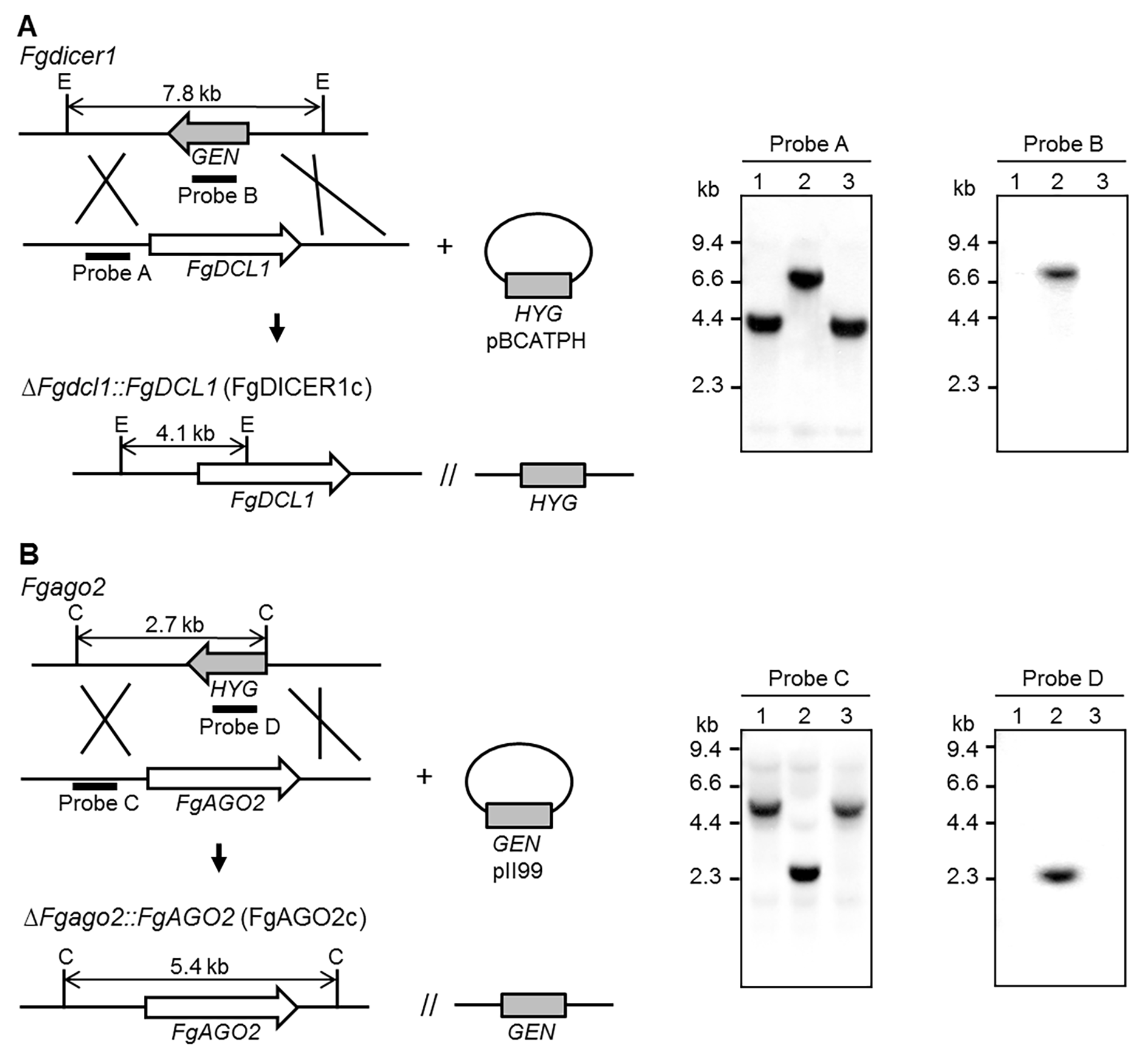

Supplement: S2 Fig — Complementation experiments of Fgdicer1 (A) and Fgago2 (B) were performed, and the resulting strains were confirmed by Southern blot analysis. Lane 1, F. graminearum wild-type strain Z-3636; lane 2, a deletion mutant; lane 3, a complemented strain. The sizes of the DNA standards (kb) used are indicated to the left of each blot. E, EcoRI; P, C, ClaI. (TIF) [file pgen.1006595.s002.tif]

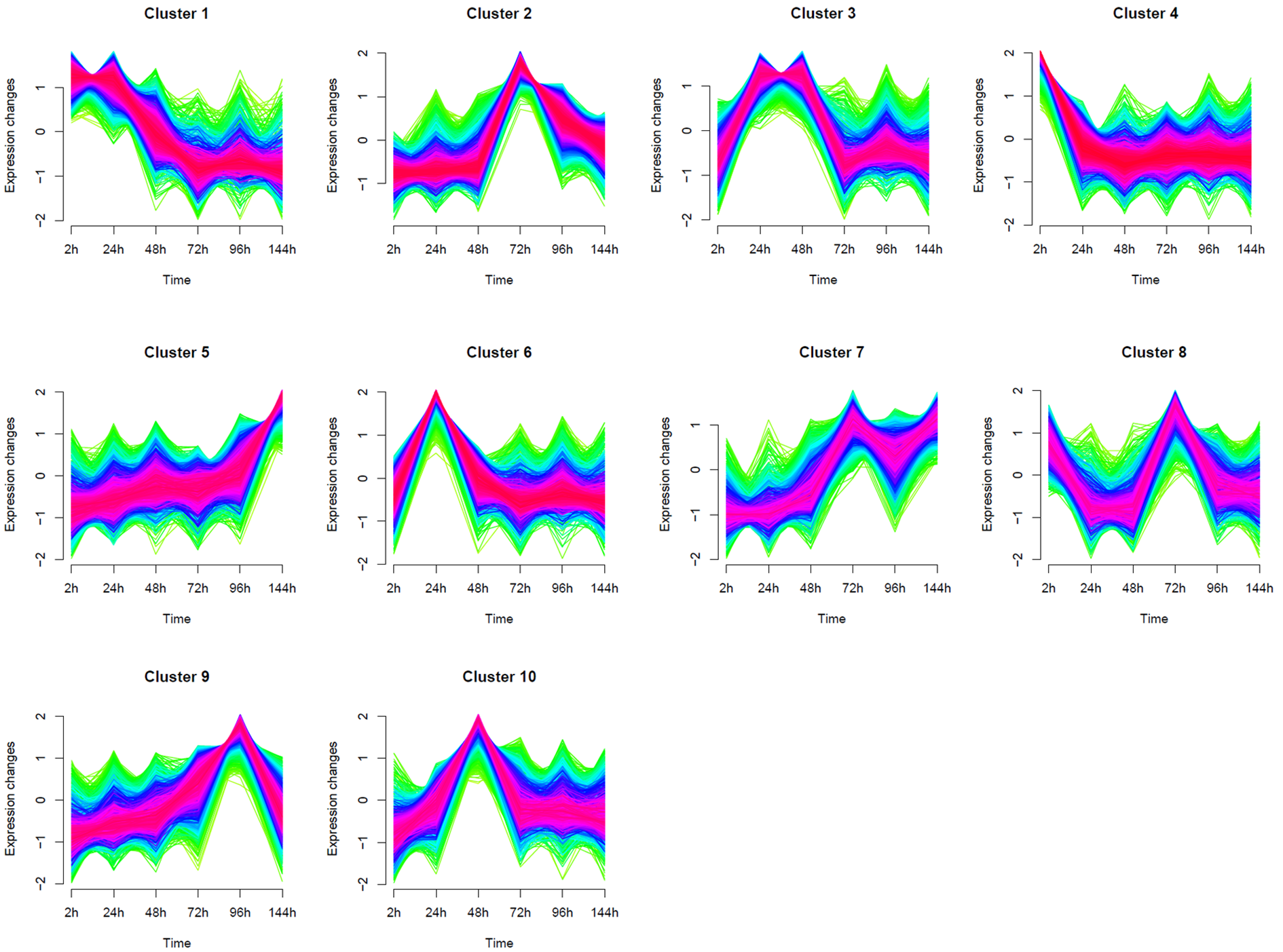

Supplement: S3 Fig — Fuzzy clustering categorized total genes into 10 groups depending on their expression profiles during sexual development. RNA-seq results were obtained from a previous study and realigned for this analysis [8]. (TIF) [file pgen.1006595.s003.tif]

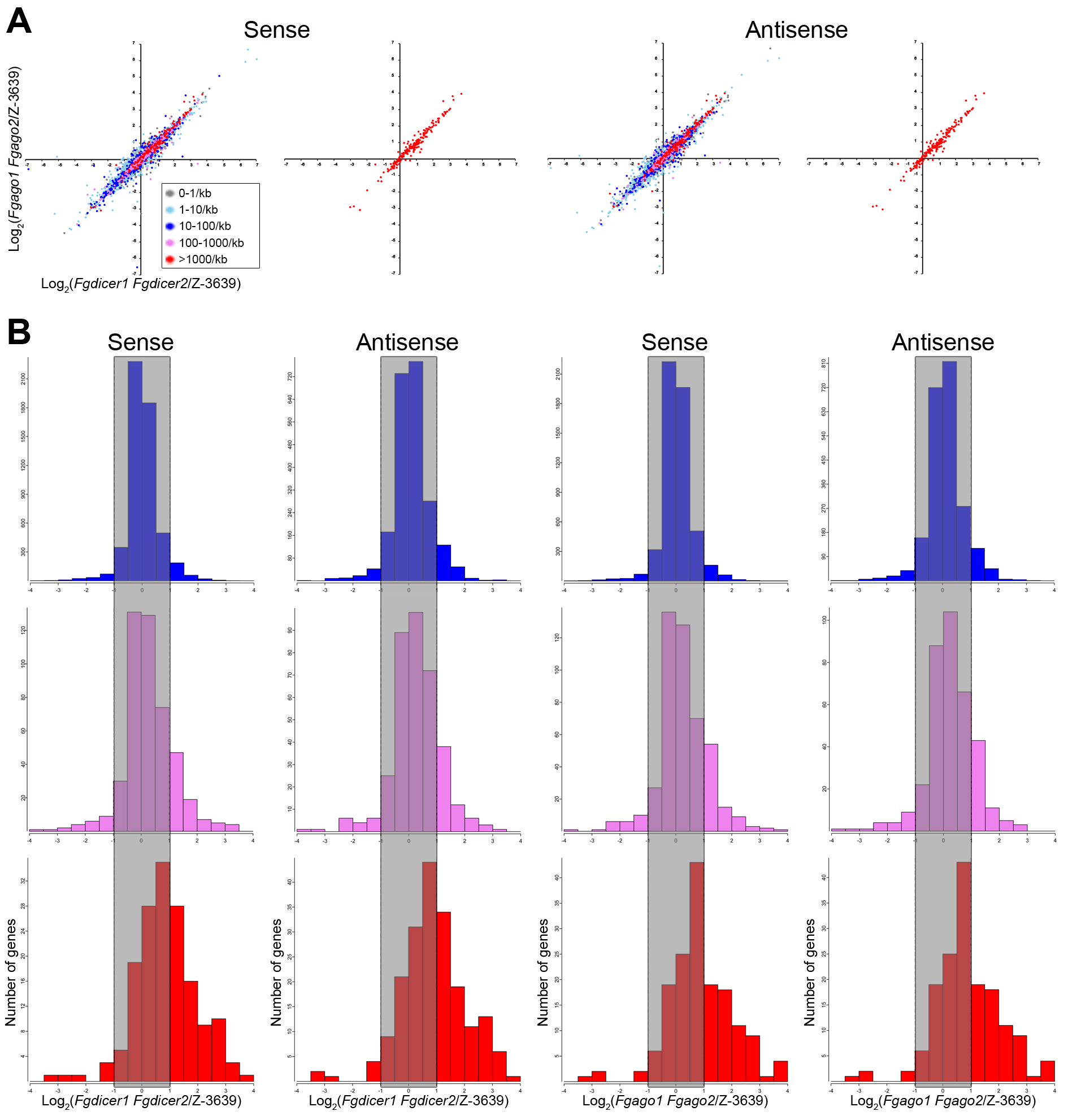

Supplement: S4 Fig — (A) Correlation analyses of transcriptomes of Fgdicer1 Fgdicer2 and Fgago1 Fgago2 compared to the wild type depending on counts of 5′-U sRNAs (22–25 nt). The log2 ratio of transcript abundance in Fgdicer1 Fgdicer2 versus wild-type (x axis) and Fgago1 Fgago2 versus wild-type (y axis) is plotted. Colors indicate the sRNA density (reads per kilobase). (B) Correlation analyses between sRNA counts and transcript abundance. Gene numbers with corresponding log2 ratio of transcript abundance in Fgdicer1 Fgdicer2 or Fgago1 Fgago2 versus the wild-type strain Z-3639 were counted. Most genes producing antisense sRNAs at more than 1000 counts per kilobase (red graphs) were positively regulated in Fgdicer1 Fgdicer2 and Fgago1 Fgago2 compared to the wild type. Colors indicate the sRNA density. (TIF) [file pgen.1006595.s004.tif]

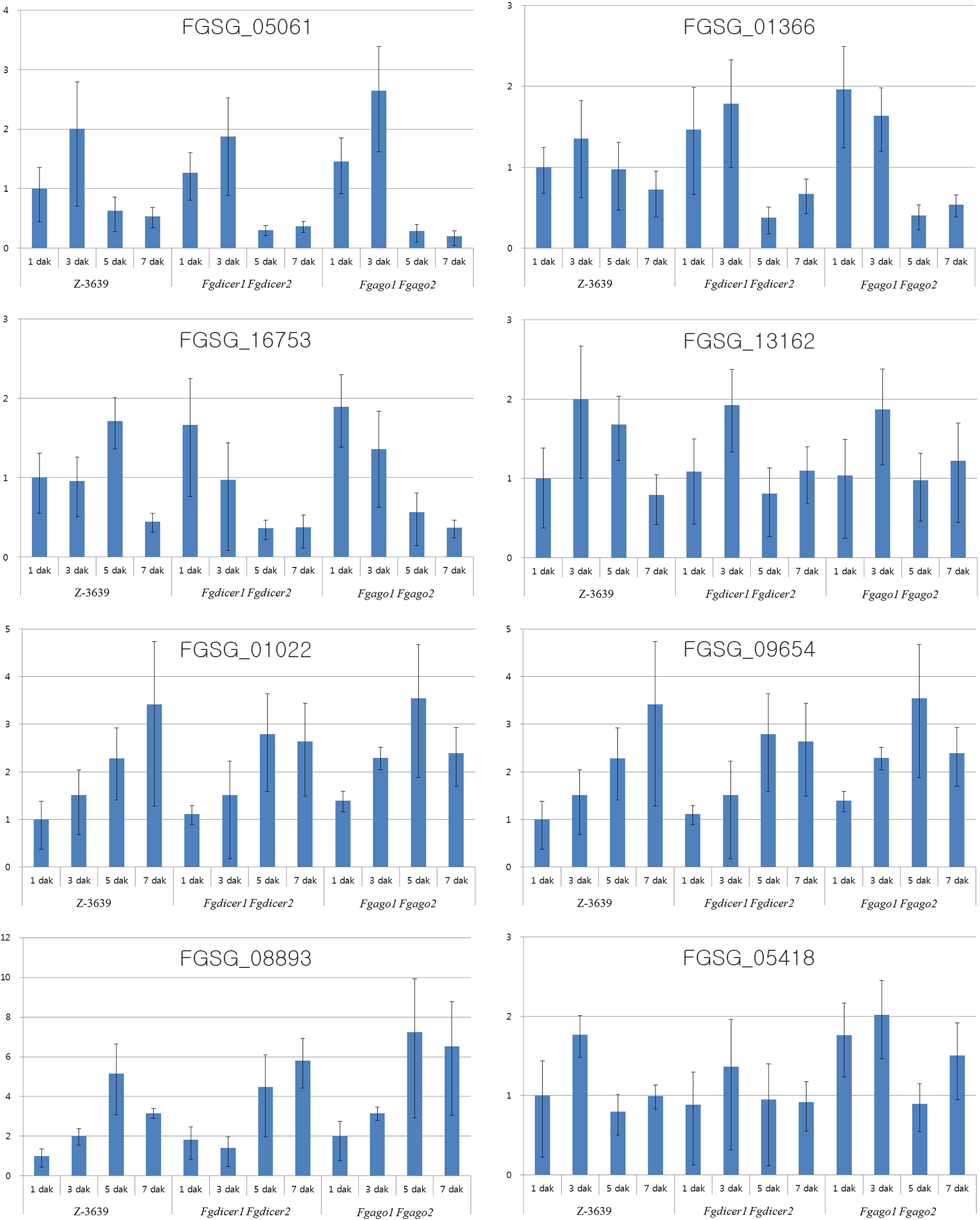

Supplement: S5 Fig — The transcript levels of each locus were analyzed by qRT-PCR in the wild-type, Fgdicer1 Fgdicer2, and Fgago1 Fgago2 strains during the sexual stages (S1, S3, S5 and S7. 1, 3, 5, and 7 days after sexual induction, respectively) on carrot agar. (TIF) [file pgen.1006595.s005.tif]

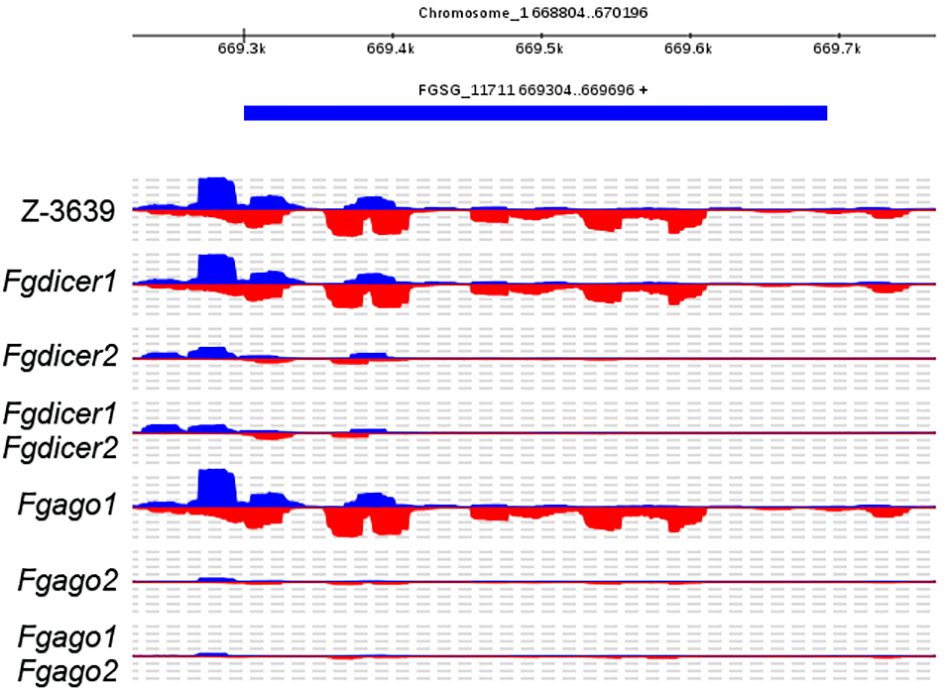

Supplement: S6 Fig — Aligned sRNA-seq results of F. graminearum strains were visualized using IGV. (TIF) [file pgen.1006595.s006.tif]

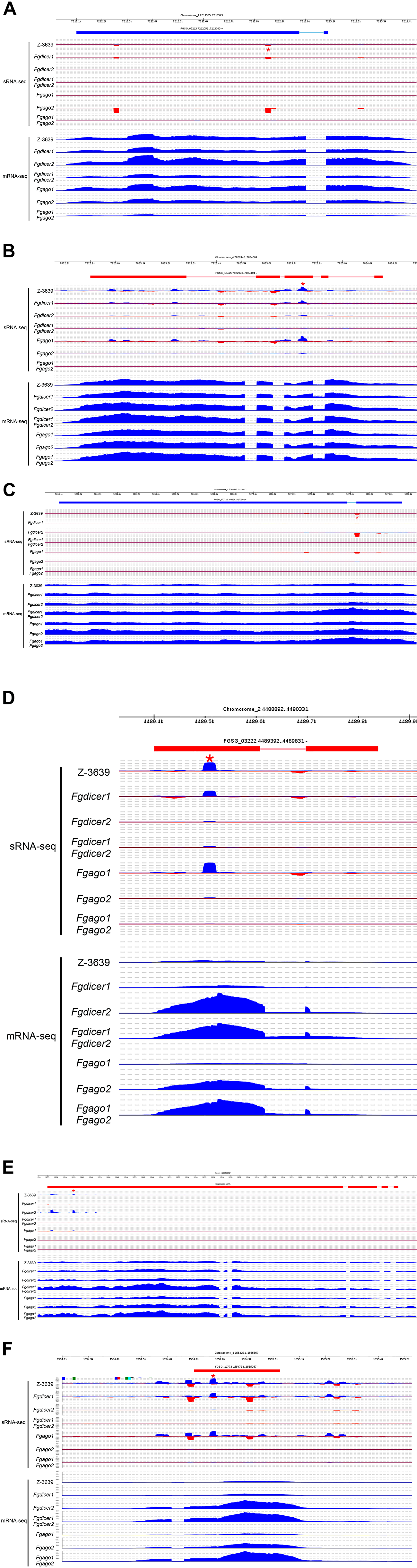

Supplement: S7 Fig — Aligned sRNA-seq results of F. graminearum strains were visualized using IGV. (TIF) [file pgen.1006595.s007.tif]

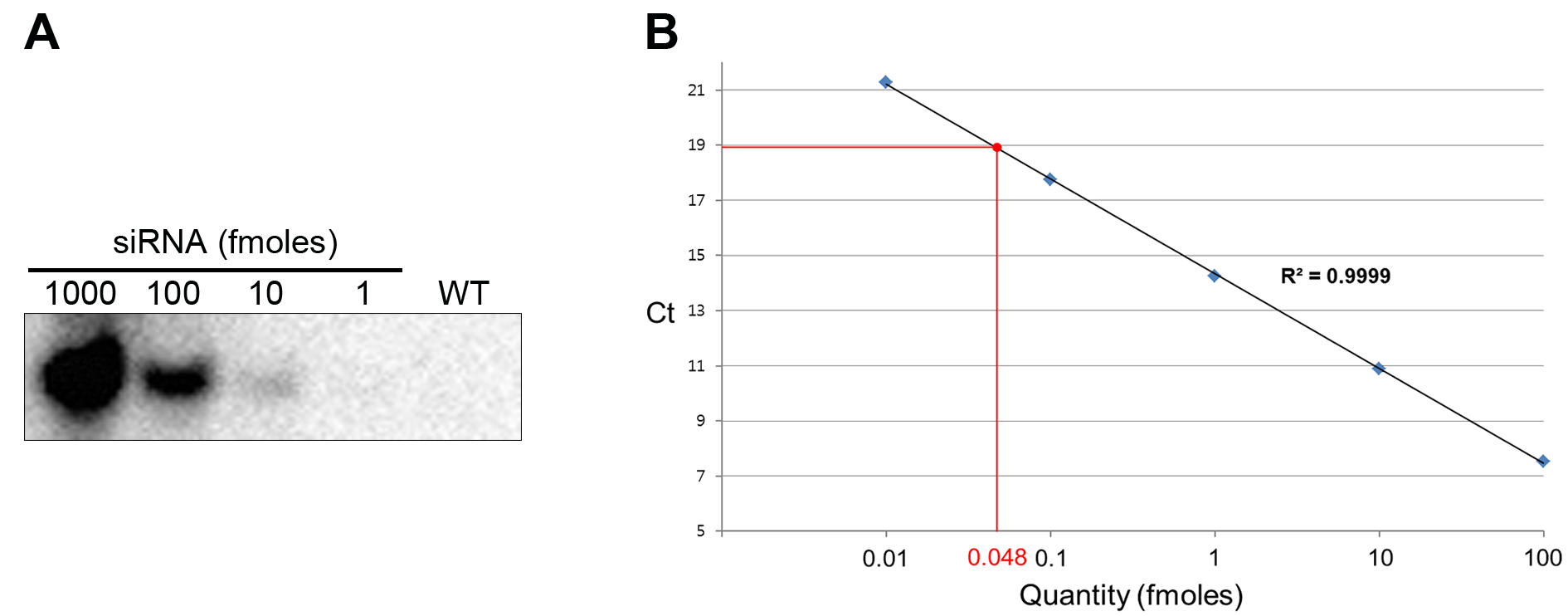

Supplement: S8 Fig — (A) Gel blot analysis of siRNA of FGSG_09213 expression. Synthetic siRNA and 1 μg of the F. graminearum wild-type total RNA (WT) enriched in small RNA species were used to determine sensitivity of the Northern blot. (B) Standard curve of the siRNA. Stem-loop RT-PCR assay was used for the analysis. Red line indicates that 0.048 fmoles of siRNA were detected in 100 ng of the small RNA-enriched RNA samples of the F. graminearum wild-type strain. (TIF) [file pgen.1006595.s008.tif]
