# Supplementary material for: Genome-wide exonic small interference RNA-mediated gene silencing regulates sexual reproduction in the homothallic fungus Fusarium graminearum
Source: PLoS Genet. 2017 Feb 1;13(2):e1006595. doi: 10.1371/journal.pgen.1006595 (PMC5310905; doi:10.1371/journal.pgen.1006595)
Supplement: S1 Table — (DOC) [file pgen.1006595.s009.doc]

**S1 Table. *F. graminearum* strains used in this study.**

| **Strain** | **Genotype** | **Reference or parents** |
| --- | --- | --- |
| Z-3639 | *F. graminearum* wild-type |  |
| Δ*mat2* | *∆mat1-2::GFP-HYG* |  |
| HK334 | *∆Fgdicer1::GEN* | Z-3639 |
| HK335 | *∆Fgdicer2::HYG* | Z-3639 |
| HK336 | *∆Fgago1::GEN* | Z-3639 |
| HK337 | *∆Fgago2::HYG* | Z-3639 |
| HK338 | *∆mat1-2::GFP-HYG ∆Fgdicer2::HYG* | Δ*mat2* × HK335 |
| HK339 | *∆mat1-2::GFP-HYG ∆Fgago1::GEN* | Δ*mat2* × HK336 |
| HK340 | *∆Fgdicer1::GEN ∆Fgdicer2::HYG* | HK338 × HK334 |
| HK341 | *∆Fgago1::GEN ∆Fgago2::HYG* | HK339 × HK337 |
| HK342 | *∆Fgdicer1::FgDICER1 pBCATPH* (*HYG*) | HK334 |
| HK343 | *∆Fgago2::FgAGO2-HYG pII99* (*GEN*) | HK337 |
