# Supplementary material for: Genome-wide exonic small interference RNA-mediated gene silencing regulates sexual reproduction in the homothallic fungus Fusarium graminearum
Source: PLoS Genet. 2017 Feb 1;13(2):e1006595. doi: 10.1371/journal.pgen.1006595 (PMC5310905; doi:10.1371/journal.pgen.1006595)
Supplement: S4 Table — (DOC) [file pgen.1006595.s012.doc]

**S4 Table. Number of differentially expressed genes depending on cluster groups.**

| Cluster | Total number | Upregulated genes | Downregulated genes |
| --- | --- | --- | --- |
| 1 | 1123 | 9 | 2 |
| 2 | 1703 | 51 | 4 |
| 3 | 1237 | 11 | 5 |
| 4 | 1285 | 10 | 2 |
| 5 | 1378 | 3 | 42 |
| 6 | 1356 | 16 | 13 |
| 7 | 1202 | 9 | 4 |
| 8 | 859 | 1 | 2 |
| 9 | 1382 | 61 | 22 |
| 10 | 1221 | 15 | 14 |
