# Supplementary material for: Genome-wide exonic small interference RNA-mediated gene silencing regulates sexual reproduction in the homothallic fungus Fusarium graminearum
Source: PLoS Genet. 2017 Feb 1;13(2):e1006595. doi: 10.1371/journal.pgen.1006595 (PMC5310905; doi:10.1371/journal.pgen.1006595)
Supplement: S5 Table — (DOC) [file pgen.1006595.s013.doc]

**S5 Table. Statistical summary of sRNA sequencing raw data.**

|  |  | Z-3639 | *Fgdicer1* | *Fgdicer2* | *Fgdicer1 Fgdicer2* | *Fgago1* | *Fgago2* | *Fgago1 Fgago2* |
| --- | --- | --- | --- | --- | --- | --- | --- | --- |
| Total reads | Total | 46,536,421 | 49,693,560 | 68,218,805 | 47,625,408 | 65,887,967 | 56,256,557 | 40,566,362 |
|  | Collapsed | 8,443,648 | 7,329,439 | 10,251,225 | 11,274,763 | 10,227,209 | 9,314,330 | 9,056,743 |
| Total reads (18-32 nt) | Total | 29,432,024 | 29,857,338 | 38,650,541 | 24,944,339 | 44,464,666 | 30,515,345 | 23,216,204 |
|  | Collapsed | 5,949,081 | 5,076,267 | 6,534,746 | 5,765,324 | 7,625,532 | 5,605,655 | 5,633,340 |
| Masked reads  (rRNA, tRNA, mtRNA) | Total | 19,095,495 | 14,455,648 | 17,090,569 | 12,548,234 | 30,317,657 | 14,545,568 | 11,767,589 |
|  | Collapsed | 5,660,502 | 4,803,168 | 6,170,782 | 5,475,729 | 7,343,591 | 5,272,501 | 5,354,053 |
| Mapped reads | Total | 12,993,604 | 9,847,638 | 13,342,406 | 10,127,725 | 20,866,230 | 10,252,104 | 8,206,012 |
|  | Collapsed | 3,607,807 | 3,168,612 | 4,873,927 | 4,534,751 | 4,599,469 | 4,201,702 | 3,998,097 |
