# Supplementary material for: Genome-wide exonic small interference RNA-mediated gene silencing regulates sexual reproduction in the homothallic fungus Fusarium graminearum
Source: PLoS Genet. 2017 Feb 1;13(2):e1006595. doi: 10.1371/journal.pgen.1006595 (PMC5310905; doi:10.1371/journal.pgen.1006595)
Supplement: S6 Table — (DOC) [file pgen.1006595.s014.doc]

**S6 Table. Statistical summary of aligned sRNA sequencing data.**

|  |  | Z-3639 | *Fgdicer1* | *Fgdicer2* | *Fgdicer1 Fgdicer2* | *Fgago1* | *Fgago2* | *Fgago1 Fgago2* |
| --- | --- | --- | --- | --- | --- | --- | --- | --- |
| Exon | sense | 5251547 (51.1)^a^ | 5553969 (49.3) | 4114321 (31.3) | 4743003 (26.5) | 5650676 (55.4) | 3919287 (31.4) | 4100041 (28.2) |
|  | antisense | 3482982 (87.8) | 2133569 (82.3) | 1507654 (84.1) | 229036 (20.8) | 4247473 (86.9) | 720738 (85.3) | 294684 (53.9) |
| Intron | sense | 631834 (74.1) | 592325 (72.6) | 295184 (50.7) | 198560 (42.6) | 764432 (76.1) | 256863 (54.4) | 167163 (45.4) |
|  | antisense | 608826 (86.8) | 354174 (81.6) | 365406 (85.0) | 42028 (17.3) | 754160 (87.2) | 91201 (73.2) | 43747 (40.6) |
| 5′ UTR | sense | 1288712 (75.1) | 1094581 (71.2) | 556352 (48.2) | 412958 (32.5) | 1548271 (76.6) | 454412 (51.2) | 367400 (41.4) |
|  | antisense | 1429868 (82.8) | 1097007 (77.2) | 419906 (65.7) | 155727 (31.4) | 1810717 (82.8) | 287027 (65.0) | 167587 (47.9) |
| 3′ UTR | sense | 1277981 (68.6) | 1166303 (66.0) | 757300 (48.5) | 595469 (31.1) | 1445697 (70.4) | 646412 (44.1) | 511497 (34.6) |
|  | antisense | 1132136 (82.6) | 794885 (77.6) | 551804 (72.5) | 155758 (27.9) | 1325455 (82.0) | 343872 (73.5) | 169986 (47.3) |
| Intergenic |  | 3655587 (67.4) | 3611950 (58.8) | 2328201 (47.8) | 1051076 (30.6) | 4260009 (70.3) | 1399121 (40.6) | 976412 (34.4) |
| rRNA | sense | 12194240 (25.4) | 20144014 (27.1) | 15659188 (25.1) | 8894948 (24.7) | 12144503 (24.4) | 11684779 (25.6) | 8708867 (23.9) |
|  | antisense | 37229 (86.9) | 6917 (69.4) | 75233 (87.7) | 1882 (15.4) | 43736 (85.9) | 15176 (86.4) | 4490 (54.7) |
| tRNA | sense | 1547630 (60.8) | 1789195 (66.2) | 1591150 (67.9) | 1631709 (70.1) | 1452148 (56.5) | 1521966 (68.3) | 1125330 (64.6) |
|  | antisense | 162662 (93.1) | 12938 (73.1) | 384267 (94.0) | 2639 (20.4) | 220418 (92.8) | 16272 (94.4) | 3087 (64.8) |

^a^ The percentage of counts of small RNAs with 5′ U is given in parentheses.
