# Supplementary material for: Genome-wide exonic small interference RNA-mediated gene silencing regulates sexual reproduction in the homothallic fungus Fusarium graminearum
Source: PLoS Genet. 2017 Feb 1;13(2):e1006595. doi: 10.1371/journal.pgen.1006595 (PMC5310905; doi:10.1371/journal.pgen.1006595)
Supplement: S8 Table — (DOC) [file pgen.1006595.s016.doc]

**S8 Table. Characterization of sRNA-producing genes.**

|  | **Z-3639** | | ***Fgdicer1*** | | ***Fgdicer2*** | | ***Fgdicer1 Fgdicer2*** | | ***Fgago1*** | | ***Fgago2*** | | ***Fgago1 Fgago2*** | |
| --- | --- | --- | --- | --- | --- | --- | --- | --- | --- | --- | --- | --- | --- | --- |
|  | **Total** | **5′-U** | **Total** | **5′-U** | **Total** | **5′-U** | **Total** | **5′-U** | **Total** | **5′-U** | **Total** | **5′-U** | **Total** | **5′-U** |
| Total | 5,970 | 1,088 | 6,068 | 760 | 5,935 | 481 | 5,742 | 6 | 5,995 | 1,074 | 5,486 | 465 | 5,438 | 166 |
| Sense | 4,820 | 98 | 5,240 | 109 | 5,133 | 34 | 5,369 | 2 | 4,854 | 102 | 4,855 | 26 | 5,005 | 31 |
| Antisense | 149 | 448 | 102 | 186 | 148 | 404 | 106 | 4 | 142 | 379 | 136 | 304 | 102 | 130 |
| Both strands | 1,001 | 542 | 726 | 465 | 654 | 43 | 267 | 0 | 999 | 593 | 495 | 135 | 331 | 5 |
