# Supplementary material for: Genome-wide exonic small interference RNA-mediated gene silencing regulates sexual reproduction in the homothallic fungus Fusarium graminearum
Source: PLoS Genet. 2017 Feb 1;13(2):e1006595. doi: 10.1371/journal.pgen.1006595 (PMC5310905; doi:10.1371/journal.pgen.1006595)
Supplement: S10 Table — (DOC) [file pgen.1006595.s018.doc]

**S10 Table. Size distribution of adaptor-trimmed degradome sequencing raw reads.**

| Size (nt) | Library 1 | Library 2 |
| --- | --- | --- |
| 12 | 1,229,715 | 750,906 |
| 13 | 582,663 | 395,909 |
| 14 | 279,501 | 207,678 |
| 15 | 2,084,898 | 2,038,479 |
| 16 | 86,322,838 | 93,098,799 |
| 17 | 69,375,281 | 77,280,267 |
| 18 | 101,006 | 72,984 |
| 19 | 35,559 | 13,720 |
| 20 | 28,867 | 9,335 |
| 21 | 26,618 | 8,058 |
| 22 | 21,311 | 4,827 |
| 23 | 19,768 | 3,900 |
| 24 | 208,811 | 56,168 |
| 25 | 179,887 | 37,226 |
| 26 | 11,770 | 1,274 |
| 27 | 15,267 | 1,557 |
| 28 | 10,472 | 1,045 |
| 29 | 6,146 | 504 |
| 30 | 13,346 | 1,238 |
