# Supplementary material for: Genome-wide exonic small interference RNA-mediated gene silencing regulates sexual reproduction in the homothallic fungus Fusarium graminearum
Source: PLoS Genet. 2017 Feb 1;13(2):e1006595. doi: 10.1371/journal.pgen.1006595 (PMC5310905; doi:10.1371/journal.pgen.1006595)
Supplement: S11 Table — (DOC) [file pgen.1006595.s019.doc]

**S11 Table. Statistical summary of degradome sequencing results and distribution of degradome tags mapped to the genome sequence of *F. graminearum*.**

|  |  | Library 1 | Library 2 |
| --- | --- | --- | --- |
| Total reads (12-30 nt) |  | 160,553,724 | 173,983,874 |
| Total reads (16-17 nt) |  | 155,698,119 | 170,379,066 |
| Mapped reads |  | 148,013,319 | 161,960,891 |
| Exon | Sense | 74,497,181 | 82,457,072 |
|  | Antisense | 3,895,123 | 4,253,725 |
| Intron | Sense | 1,056,291 | 1,109,561 |
|  | Antisense | 650,170 | 719,344 |
| 3’UTR  (0~100 bp) | Sense | 24,435,836 | 26,522,911 |
|  | Antisense | 1,139,488 | 1,198,118 |
| 3’UTR  (~200 bp) | Sense | 14,874,096 | 16,215,819 |
|  | Antisense | 1,661,492 | 1,801,172 |
| 3’UTR  (~300 bp) | Sense | 6,863,947 | 7,597,671 |
|  | Antisense | 899,877 | 980,227 |
| 3’UTR  (~400 bp) | Sense | 3,333,971 | 3,663,066 |
|  | Antisense | 583,064 | 603,672 |
| 3’UTR  (~500 bp) | Sense | 1,689,533 | 1,838,648 |
|  | Antisense | 494,479 | 533,969 |
| 5’ UTR  (~ 500 bp) | Sense | 3,603,256 | 3,513,184 |
|  | Antisense | 1,169,860 | 1,220,473 |
| Intergenic |  | 7,165,656 | 7,732,258 |
