# Supplementary material for: Genome-wide exonic small interference RNA-mediated gene silencing regulates sexual reproduction in the homothallic fungus Fusarium graminearum
Source: PLoS Genet. 2017 Feb 1;13(2):e1006595. doi: 10.1371/journal.pgen.1006595 (PMC5310905; doi:10.1371/journal.pgen.1006595)
Supplement: S13 Table — (DOC) [file pgen.1006595.s021.doc]

**S13 Table. Primers used in this study.**

| **Primer** | **Sequence (5’ → 3’)** | **Description** |
| --- | --- | --- |
| FgDICER1-5F | ATTGGCAAGGCATGGTACTCTGAG | Forward and reverse primers for amplification of 5’ flanking region of *FgDICER1* with tail for the geneticin resistance gene cassette fusion |
| FgDICER1-5R | gcacaggtacacttgtttagagaGAGTCGGTGTCCATATTGAGGCTGTA |
| FgDICER1-3F | ccttcaatatcatcttctgtcgaACAGCTGTCTAAGTACTGCAGGGTA | Forward and reverse primers for amplification of 3’ flanking region of *FgDICER1* with tail for geneticin resistance gene cassette fusion |
| FgDICER1-3R | ATGATGTTCGCACAGGGATATGCTCGT |
| FgDICER1-5N | GTAGCTCAGCTATGCAGAACAGTCGA | Forward and reverse nest primers for third fusion PCR for amplification of *FgDICER1* deletion construct |
| FgDICER1-3N | AGCGAGGACTCAATGGACTCTTTCGT |
| FgDICER2-5F | TGTCTCCATCGACTTCGACGTAGCT | Forward and reverse primers for amplification of 5’ flanking region of *FgDICER2* with tail for the hygromycin resistance gene cassette fusion |
| FgDICER2-5R | tgagacaaatggtgttcaggatctcCTCAGTTTGTGCAAGATAGGTCGCA |
| FgDICER2-3F | ctgtgtgaaattgttatccgctcacGTTGCCATGGAACTCATTTAGTTGGAG | Forward and reverse primers for amplification of 3’ flanking region of *FgDICER2* with tail for hygromycin resistance gene cassette fusion |
| FgDICER2-3R | GAAGAGACATGTGAGGCCAAGACAGAT |
| FgDICER2-5N | ACGCATACCTTGACTCGTCAACTTCG | Forward and reverse nest primers for third fusion PCR for amplification of *FgDICER2* deletion construct |
| FgDICER2-3N | ACTGACGGCCTGTTCCATGCGAATGTA |
| FgAGO1-5F | AGGCTTGGATGCATCGAATTTGGCTCTCT | Forward and reverse primers for amplification of 5’ flanking region of *FgAGO1* with tail for the geneticin resistance gene cassette fusion |
| FgAGO1-5R | gcacaggtacacttgtttagagaATGTCTGGGTACTCAAAGAATGGGCCA |
| FgAGO1-3F | ccttcaatatcatcttctgtcgaTAAGTGTGCCGTGTGTGGGAGATGAGTT | Forward and reverse primers for amplification of 3’ flanking region of *FgAGO1* with tail for geneticin resistance gene cassette fusion |
| FgAGO1-3R | TCAACCTCGTTCCGTTCAACTCGATACA |
| FgAGO1-5N | 5'-TGGGCTACATACGAAGCAACCTCTCCA-3' | Forward and reverse nest primers for third fusion PCR for amplification of *FgAGO1* deletion construct |
| FgAGO1-3N | 5'-AGCTTCACATATACGCGAATACGCTCGT-3' |
| FgAGO2-5F | ACATCCTACCGTCGTCGCAAAGCTATGA | Forward and reverse primers for amplification of 5’ flanking region of *FgAGO2* with tail for the hygromycin resistance gene cassette fusion |
| FgAGO2-5R | tgagacaaatggtgttcaggatctcAGAAGTTAGTCGATGTGTGCGCGTGTGT |
| FgAGO2-3F | ctgtgtgaaattgttatccgctcacCAGAACAATGGCGTTGATGCAAGGTTTGA | Forward and reverse primers for amplification of 3’ flanking region of *FgAGO2* with tail for hygromycin resistance gene cassette fusion |
| FgAGO2-3R | ATGGGCTTGTGGACAGGATGACAGTT |
| FgAGO2-5N | ATACACGACAACCATCGGGAGAGTTTGT | Forward and reverse nest primers for third fusion PCR for amplification of *FgAGO2* deletion construct |
| FgAGO2-3N | AGTCTGGACAGGATAGGCACTGGACAT |
| Gen-F | CGACAGAAGATGATATTGAAGG | Forward and reverse primers for amplification of the geneticin cassette from the pII99 vector |
| Gen-R | CTCTAAACAAGTGTACCTGTG |
| Hyg-F | GAGATCCTGAACACCATTTGTCTCA | Forward and reverse primers for amplification of the hygromycin cassette from the pBCATPH vector |
| Hyg-R | GTGAGCGGATAACAATTTCACACAG |
| FgDICER1-NF | ACAACACACTTGGACTACCAGACGA | Forward and reverse primers for real-time PCR of *FgDICER1* |
| FgDICER1-NR | TCTGCTTGGTCTCCTCTTGCCAACA |
| FgDICER2-NF | ACGACGAAATCCTTGAGCTCGAGGT | Forward and reverse primers for real-time PCR of *FgDICER2* |
| FgDICER2-NR | TGATGTCCATCGCCGAGTACTCAGA |
| FgAGO1-NF | AAGATATCCGGTAACTGTGCCAAGCT | Forward and reverse primers for real-time PCR of *FgAGO1* |
| FgAGO1-NR | ACGACAGGCTCGTTCAATCTCAGGT |
| FgAGO2-NF | AGATCGGTCAAGATCCTTATCTGAG | Forward and reverse primers for real-time PCR of *FgAGO2* |
| FgAGO2-NR | TGGTCTTGAATGTTGTGGCGAATGCT |
| 05161-NF | CCAGCCATGCTGGAAGGAGA | Forward and reverse primers for real-time PCR of *FGSG_05161* |
| 05161-NR | TTAGCCTCGCGCTTAGCCAT |
| 01366-NF | CTACCGCCTTTGATGCCGTT | Forward and reverse primers for real-time PCR of *FGSG_01366* |
| 01366-NR | ACTGGCGCATACTCATGCCA |
| 16753-NF | ACCCGAGCGTACTGTCCAAG | Forward and reverse primers for real-time PCR of *FGSG_16753* |
| 16753-NR | ACGTTCGCGACACTTGATGC |
| 13162-NF | TGGCAACTAGCCGGCCTAAA | Forward and reverse primers for real-time PCR of *FGSG_13162* |
| 13162-NR | TCTTCTTCGCCACTGTCGCT |
| 01022-NF | TGCCCATGATCTCGGCCATA | Forward and reverse primers for real-time PCR of *FGSG_01022* |
| 01022-NR | CTTGTCGGGCAACTCGGTTT |
| 09654-NF | GCTGAGGCTGCCCTGACA | Forward and reverse primers for real-time PCR of *FGSG_09654* |
| 09654-NR | CGCGCTTGATCCAGATGGTG |
| 08893-NF | TGGCAGACGACATTAAGGAGGAGCAC | Forward and reverse primers for real-time PCR of *FGSG_08893* (*MAT1-2-1*) |
| 08893-NR | TGAGCAGCGACAGCAGCAGCAAGAA |
| 05418-NF | GCAATCTCGACGCTGCCTTC | Forward and reverse primers for real-time PCR of *FGSG_05418* |
| 05418-NR | GATTTGCGCGACGAAAGGGT |
| 09213_siRNA probe | CTTCATCACAACAATGCCAGA | Northern probe for siRNA of FGSG_09213 |
| 09213_siRNA-RT | GTCGTATCCAGTGCAGGGTCCGAGGTATTCGCACTGGATACGACCTTCAT | RT stem-loop primer for RT-PCR of FGSG_09213 siRNA |
| 09213_siRNA-F | TGCCCGTTCTGGCATTGTTGTG | Forward primer for RT-PCR of FGSG_09213 siRNA |
| 13495_siRNA-RT | GTCGTATCCAGTGCAGGGTCCGAGGTATTCGCACTGGATACGACTAATCC | RT stem-loop primer for RT-PCR of FGSG_13495 siRNA |
| 13495_siRNA-F | GCTATGAGATTCGTGCCCTCTGT | Forward primer for RT-PCR of FGSG_13495 siRNA |
| 17272_siRNA-RT | GTCGTATCCAGTGCAGGGTCCGAGGTATTCGCACTGGATACGACTACGCA | RT stem-loop primer for RT-PCR of FGSG_17272 siRNA |
| 17272_siRNA-F | GTCTCAAGCAAGAGTTCTGCGG | Forward primer for RT-PCR of FGSG_17272 siRNA |
| 03222_siRNA-RT | GTCGTATCCAGTGCAGGGTCCGAGGTATTCGCACTGGATACGACAAAGAG | RT stem-loop primer for RT-PCR of FGSG_03222 siRNA |
| 03222_siRNA-F | GGGCGCCAGAGGTCGA | Forward primer for RT-PCR of FGSG_03222 siRNA |
| 10502_siRNA-RT | GTCGTATCCAGTGCAGGGTCCGAGGTATTCGCACTGGATACGACGGGTGA | RT stem-loop primer for RT-PCR of FGSG_10502 siRNA |
| 10502_siRNA-F | TCGGCCGGGGACTCG | Forward primer for RT-PCR of FGSG_10502 siRNA |
| 11773_siRNA-RT | GTCGTATCCAGTGCAGGGTCCGAGGTATTCGCACTGGATACGACTATAGC | RT stem-loop primer for RT-PCR of FGSG_11773 siRNA |
| 11773_siRNA-F | GGCGTGCAAAGAACTACTCGAC | Forward primer for RT-PCR of FGSG_11773 siRNA |
| siRNA-R | GTGCAGGGTCCGAGGT | Common reverse primer for RT-PCR of siRNA |
| 10502-NF | TGTCACCATCAGCGGCTTCT | Forward and reverse primers for real-time PCR of *FGSG_10502* |
| 10502-NR | AGGGTTGGTAACCGGAGCAG |
| 03222-NF | CCTCAACCGAGCCATCCGA | Forward and reverse primers for real-time PCR of *FGSG_03222* |
| 03222-NR | ACTGCAGAAGGCGGATCCAA |
